# Acquisition of chromosome 1q duplication in parental and genome‐edited human‐induced pluripotent stem cell‐derived neural stem cells results in their higher proliferation rate in vitro and in vivo

**DOI:** 10.1111/cpr.12892

**Published:** 2020-09-12

**Authors:** Narges Zare Mehrjardi, Marek Molcanyi, Firuze Fulya Hatay, Marco Timmer, Ebrahim Shahbazi, Justus P. Ackermann, Stefan Herms, Stefanie Heilmann‐Heimbach, Thomas F. Wunderlich, Nora Prochnow, Aiden Haghikia, Angelika Lampert, Jürgen Hescheler, Edmund A. M. Neugebauer, Hossein Baharvand, Tomo Šarić

**Affiliations:** ^1^ Center for Physiology and Pathophysiology Institute for Neurophysiology Medical Faculty University of Cologne Cologne Germany; ^2^ Department of Stem Cells and Developmental Biology Cell Science Research Center Royan Institute for Stem Cell Biology and Technology ACECR Tehran Iran; ^3^ Department of Neurosurgery University Hospital Cologne Cologne Germany; ^4^ Center for Molecular Medicine Cologne University of Cologne Cologne Germany; ^5^ Department of Genomics, Life & Brain Center Institute for Human Genetics University of Bonn Bonn Germany; ^6^ Department of Biomedicine Medical Genetics Research Group Genomics University Hospital Basel Basel Switzerland; ^7^ Max Planck Institute for Metabolism Research and Institute for Genetics, University of Cologne Cologne Germany; ^8^ Cologne Cluster of Excellence in Cellular Stress Responses in Aging‐Associated Diseases (CECAD) Cologne Germany; ^9^ Clinic for Neurology St. Josef‐Hospital Clinic of the Ruhr‐University Bochum Bochum Germany; ^10^ Institute of Physiology Uniklinik, RWTH Aachen University Aachen Germany; ^11^ Medizinische Hochschule Brandenburg Theodor Fontane, Campus Neuruppin Neuruppin Germany; ^12^ Department of Developmental Biology University of Science and Culture Tehran Iran; ^13^ Present address: Department of Preclinical Studies Clinic for Neurosurgery University Hospital Düsseldorf Düsseldorf Germany

**Keywords:** AAVS1, cell‐based therapy, chromosome aberrations, differentiation, duplication, genome editing, neurons, regenerative medicine, transplantation, zinc‐finger nuclease

## Abstract

**Objectives:**

Genetic engineering of human‐induced pluripotent stem cell‐derived neural stem cells (hiPSC‐NSC) may increase the risk of genomic aberrations. Therefore, we asked whether genetic modification of hiPSC‐NSCs exacerbates chromosomal abnormalities that may occur during passaging and whether they may cause any functional perturbations in NSCs in vitro and in vivo.

**Materials and Methods:**

The transgenic cassette was inserted into the AAVS1 locus, and the genetic integrity of zinc‐finger nuclease (ZFN)‐modified hiPSC‐NSCs was assessed by the SNP‐based karyotyping. The hiPSC‐NSC proliferation was assessed in vitro by the EdU incorporation assay and in vivo by staining of brain slices with Ki‐67 antibody at 2 and 8 weeks after transplantation of ZFN‐NSCs with and without chromosomal aberration into the striatum of immunodeficient rats.

**Results:**

During early passages, no chromosomal abnormalities were detected in unmodified or ZFN‐modified hiPSC‐NSCs. However, at higher passages both cell populations acquired duplication of the entire long arm of chromosome 1, dup(1)q. ZNF‐NSCs carrying dup(1)q exhibited higher proliferation rate than karyotypically intact cells, which was partly mediated by increased expression of *AKT3* located on Chr1q. Compared to karyotypically normal ZNF‐NSCs, cells with dup(1)q also exhibited increased proliferation in vivo 2 weeks, but not 2 months, after transplantation.

**Conclusions:**

These results demonstrate that, independently of ZFN‐editing, hiPSC‐NSCs have a propensity for acquiring dup(1)q and this aberration results in increased proliferation which might compromise downstream hiPSC‐NSC applications.

## Graphical abstract

Neural stem cells (NSC) derived from human induced pluripotent stem cells (hiPSC) maintain genomic stability *in vitro* during early passages, irrespective of whether they were genetically engineered using zinc finger nucleases (ZFN) or not. However, prolonged passaging both of non‐modified or ZFN‐modified clonal NSC sublines leads to acquisition of duplication of the entire long arm of chromosome 1 [dup(1)q]. This aberration increases the proliferation of hiPSC‐NSC *in vitro* which is most likely mediated by upregulation of *AKT3* located on the duplicated chromosome. Higher proliferation rate of hiPSC‐derived NSCs carrying dup(1)q is retained *in vivo* after two weeks of transplantation into the striatum of immunodeficient rats. Although monitoring of animals for two months did not reveal any tumor formation from transplanted NSCs in both experimental groups, the presence of dup(1)q may poses a tumorigenic risk at later time points and represent a significant obstacle to use of hiPSC‐NSC for research and therapy.

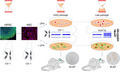

## 1 INTRODUCTION

Human‐induced pluripotent stem cell‐derived neural stem cells (hiPSC‐NSCs) have been used for developmental studies,^1^ disease modelling,^2^, ^3^ drug screening,^4^ toxicity testing^5^ and in preclinical studies of neuroregenerative therapeutic approaches.^6^ Genetic modification of stem cells is frequently utilized for lineage tracking, to modify the expression of a specific endogenous gene in order to study its biological role, or overexpress exogenous factors to monitor and/or enhance the engraftment and therapeutic efficacy of transplanted cells in regenerative approaches.^7^, ^8^, ^9^, ^10^ Genome engineering technologies such as zinc‐finger nucleases (ZFN),^11^ transcription activator‐like effector nuclease*s* (TALEN),^12^ and the clustered regularly interspaced short palindromic repeats/Cas9 (CRISPR/Cas9) system^13^, ^14^ enable DNA modifications in a highly precise manner and significantly lower the risks of various non‐target effects that are associated with traditional genetic engineering techniques.^15^ However, genome editing increases cell handling and cultivation time, which could affect their genomic stability and diminish their usefulness because the newly acquired genetic changes may be detrimental to the cell’s viability, functionality and safety.^16^, ^17^, ^18^, ^19^, ^20^

Many studies have demonstrated that different types of stem cells, including NSCs, acquire characteristic chromosomal aberrations during late and sometimes also in early passages in culture.^21^, ^22^, ^23^ Comprehensive analysis of chromosomal aberrations in 58 adult human NSC samples and 39 human embryonic stem cell (hESC)‐derived NSC samples identified a trisomy of chromosomes 7, 10, 19 and 20q as well as a trisomy and monosomy of chromosome 18.^24^ The overall frequency of aberrations in NSCs was about 9%. A similar frequency of samples with chromosomal aberrations was found in a separate analysis of hiPSC‐derived NSCs (10%, 18 out of 182 samples) and adult NSCs (7%, 7 out of 100 samples).^25^ In these samples, the most common were gains of chromosomes 1, 12 and 17, which also occur in undifferentiated human PSC cultures.^22^, ^23^, ^26^, ^27^

Targeting safe harbour areas like adeno‐associated virus site 1 (*AAVS1*) by using gene editing methods have been used in various hESC^11^, ^28^ and hiPSC^9^, ^29^ lines and their differentiated derivatives, such as NSCs.^30^, ^31^, ^32^ These studies demonstrated that cells modified by genome editing tools exhibited all properties of their parental cells and did not show perturbations in cell viability, proliferation or specialized cell functions. However, it is not fully clear whether gene editing methods increase the frequency of chromosomal aberrations in long‐term cultures, whether these aberrations cause any functional perturbations in targeted hiPSC‐NSCs and whether these functional changes would still be retained in transplanted cells in vivo.

To address these questions, we used the ZFN technology to integrate a cassette containing a human EF1‐α promoter driving the expression of puromycin resistance gene (Pac) and enhanced GFP (EPG‐cassette) into the AAVS1 locus in hiPSC‐NSCs. SNP array‐based karyotyping identified a duplication of the entire long arm of chromosome 1 [dup(1)q] in unmodified and ZFN‐modified NSCs after prolonged passaging. Compared to ZFN‐NSCs with an intact karyotype, cells that carried dup(1)q exhibited increased proliferation rate in vitro and in vivo after transplantation into the striatum of immunodeficient rats. The higher proliferation rate was partly mediated by overexpression of the proliferation promoting gene *AKT3* located in duplicated area. These results show that dup(1)q occurs in high‐passage hiPSC‐NSCs and demonstrate for the first time that its occurrence is not affected by ZFN‐based editing but that it increases cell proliferation both in vitro as well as in vivo requiring strict quality control of cells before using them for applications in research and therapy.

## 2 MATERIALS AND METHODS

### 2.1 ZFN‐mediated genome editing

The methods used for construction of the targeting vector pAAVS1‐EPG and generation of hiPSC‐NSCs are described in the Supplemental material and Figure S1. For transfection, hiPSC‐NSCs at passage 14 (p14) were treated overnight with 10 μmol/L ROCK inhibitor (Y‐27632, Selleckchem) and then dissociated with 0.05% trypsin‐EDTA (Life Technologies). Dissociated hiPSC‐NSCs (1 × 10^6^) were re‐suspended in the R buffer from the Neon Transfection System (Life Technologies) together with 8 μg of the pAAVS1‐EPG vector and 250 ng of mRNAs encoding ZFNs that target the ZFN cleavage site in the AAVS1 locus and were included in the CompoZr^®^ Targeted Integration Kit‐AAVS1 (Sigma‐Aldrich). Transfection was performed with the Neon Transfection System by using two 20 ms pulses at 1400 V. Transfected cells were plated onto a poly‐l‐ornithine/laminin‐coated (both from Sigma) 6‐well plate. Selection with 2 μg/mL puromycin began at day 7 after transfection. After 10 days, antibiotic‐resistant cells were expanded and aliquots were cryopreserved for further studies. The procedures used for generation of single cell clones and identification of mono‐ and bi‐allelic ZFN‐NSC lines are described in the Supplemental material, Figure S1, and Tables S1 and S2.

### 2.2 Molecular karyotyping

Karyotyping was performed by SNP array‐based genotyping using the Human OmniExpressExome‐8‐v1.2 BeadChip (Illumina, Inc) at the Institute for Human Genetics (Life & Brain Center, University of Bonn, Germany). Processing was performed on gDNA following the manufacturer’s procedures. Log R ratio and B‐allele frequency plots were generated in GenomeStudio V2011.1 (Illumina, Inc) using the provided manifest and cluster files, version 1.2‐B. Copy number regions were detected using the cnvPartition version 3.1.6. A visual inspection was performed for mosaicism states.

### 2.3 Cell proliferation assay

Cell proliferation assay was done by Click‐iT^®^ EdU (5‐ethynyl‐2´‐deoxyuridine) Imaging Kit (Life Technologies). Genetically non‐modified wild‐type hiPSC‐NSCs and gene‐edited ZFN‐NSCs were plated at the density of 0.1 × 10^6^/cm^2^ on poly‐l‐ornithine/laminin‐coated plates. Next day, cells were incubated with 10 µmol/L EdU for 2 hours. After fixation with 3.7% paraformaldehyde and permabilzation with 0.5% Triton X‐100, cells were detected with AlexaFluor‐555‐azide. Stained cells were analysed under Axiovert 200M microscope (Carl‐Zeiss) equipped with the image processing software Axiovision 4.5.

### 2.4 Protein and gene expression analyses

Immunocytochemical analyses, cDNA synthesis and qPCR amplification of selected genes were carried out as described in the Supplemental material. PCR primers are listed in Table S3 and antibodies in Tables S4 and S5.

### 2.5 Transplantation of ZFN‐NSCs

Animal experiments were approved by the Landesamt für Natur, Umwelt und Verbraucherschutz NRW, Recklinghausen, Germany (Permit Number: 84‐02.04.2012.A227) and conformed to the Directive 2010/63/EU of the European Parliament. Adult male Rowett Nude rats (RNU; Charles River) that weighed 250‐300 g and were 12‐14 weeks of age were anaesthetized with intraperitoneal (i.p.) injections of 60 mg/kg body weight pentobarbital. The ZFN‐NSC clone 44 without duplication (p14 + 14) and with duplication (p14 + 41) were injected into the striatum of each adult nude rat brain by a Hamilton syringe (5 µL, 33 Gauge, length: 25 mm, Pst4‐12; Hamilton) into two injection points with 0.5 × 10^5^ cells/µL per injection at the following coordinates from the bregma: striatum AP = 0.5, ML = 3.0, DV = −4.5; intraventricular AP = −0.5, ML = 1.2, DV = −3.5. Two weeks and 2 months after NSC transplantation, the animals were sacrificed and transcardially perfused with 4% paraformaldehyde. Preparation of brain tissue cryosections and immunohistochemistry procedure is described in the Supplemental Material.

### 2.6 Statistical analysis

For statistical analysis of differences between experimental groups, the independent two‐tailed Studen’s *t* test was performed by using the GraphPad Prism software (version 4.0). *P* values equal to or lower than .05 were considered statistically significant.

## 3 RESULTS

### 3.1 Generation and characterization of hiPSC‐NSCs

**Figure 1:**
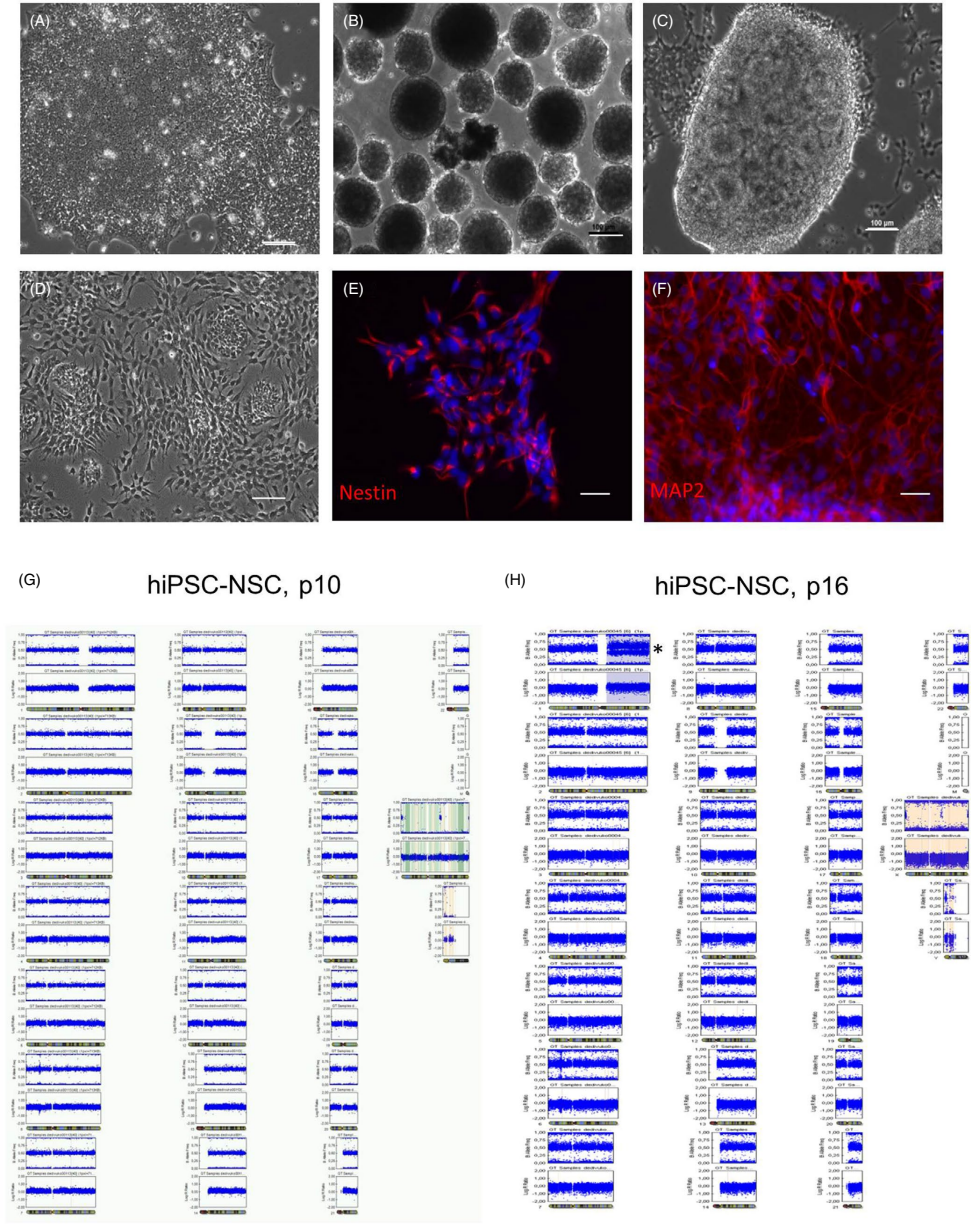
Generation and karyotype analysis of hiPSC‐NSCs. A, R‐iPSC4‐hiPSC colonies growing on Matrigel. B, Embryoid bodies (EBs) formed after digestion of hiPSCs with collagenase IV. C, Rosette‐like structures appeared 7‐10 d after plating of EBs treated with TGFβ‐inhibitor SB421543 and BMP‐inhibitor dorsomorphin onto poly‐l‐ornithine‐ and laminin‐coated plates. D, Neuroectodermal cells were obtained by dissociating rosette‐like structures and plating on poly‐l‐ornithine‐ and laminin‐coated plates. E, F, These cells expressed the NSC marker Nestin (E) and differentiated to neurons expressing microtubule‐associated protein 2 (MAP2) at day 30 of differentiation (F). Nuclei were stained with Hoechst 33342 (blue). Scale bars: 100 µm. G, H, Whole‐genome SNP array‐based karyotyping of hiPSC‐NSCs. B‐allele frequencies (upper panels) and log_2_ R ratios (lower panels) are plotted for each chromosome for all SNPs on the array located in this region. Each point is an SNP. While cells at passage 10 (p10) did not show any major karyotype abnormalities (G), hiPSC‐NSCs at p16 exhibited duplication of the entire long arm of the chromosome 1, dup(1)q (H)

Neural stem cells used in this study were derived from hiPSC line R‐iPSC4 as previously reported by us.^33^ Transcriptome, proteome and immunocytochemical analyses of these hiPSC‐NSCs revealed that they expressed typical NSC markers and exhibited robust differentiation potential to neurons, astrocytes and oligodendrocytes (see reference (33) and Figure 1A‐F). Molecular karyotyping of the parental iPSCs at passage 36 (p36) (Figure S2) and of NSCs derived from them at an early time point after their generation (p10) (Figure 1G) showed no structural chromosomal aberrations in these samples. However, after only six additional passages, hiPSC‐NSCs acquired a duplication of the entire long arm of chromosome 1 [dup(1)q] but no other abnormalities (Figure 1H, asterisk). This confirms previous reports showing that prolonged maintenance of NSCs in culture leads to the accumulation of lineage‐specific gross chromosomal aberrations in a certain fraction of cell lines.^24^, ^34^, ^35^

### 3.2 ZFN‐mediated gene targeting into the AAVS1 locus in hiPSC‐NSCs

**Figure 2:**
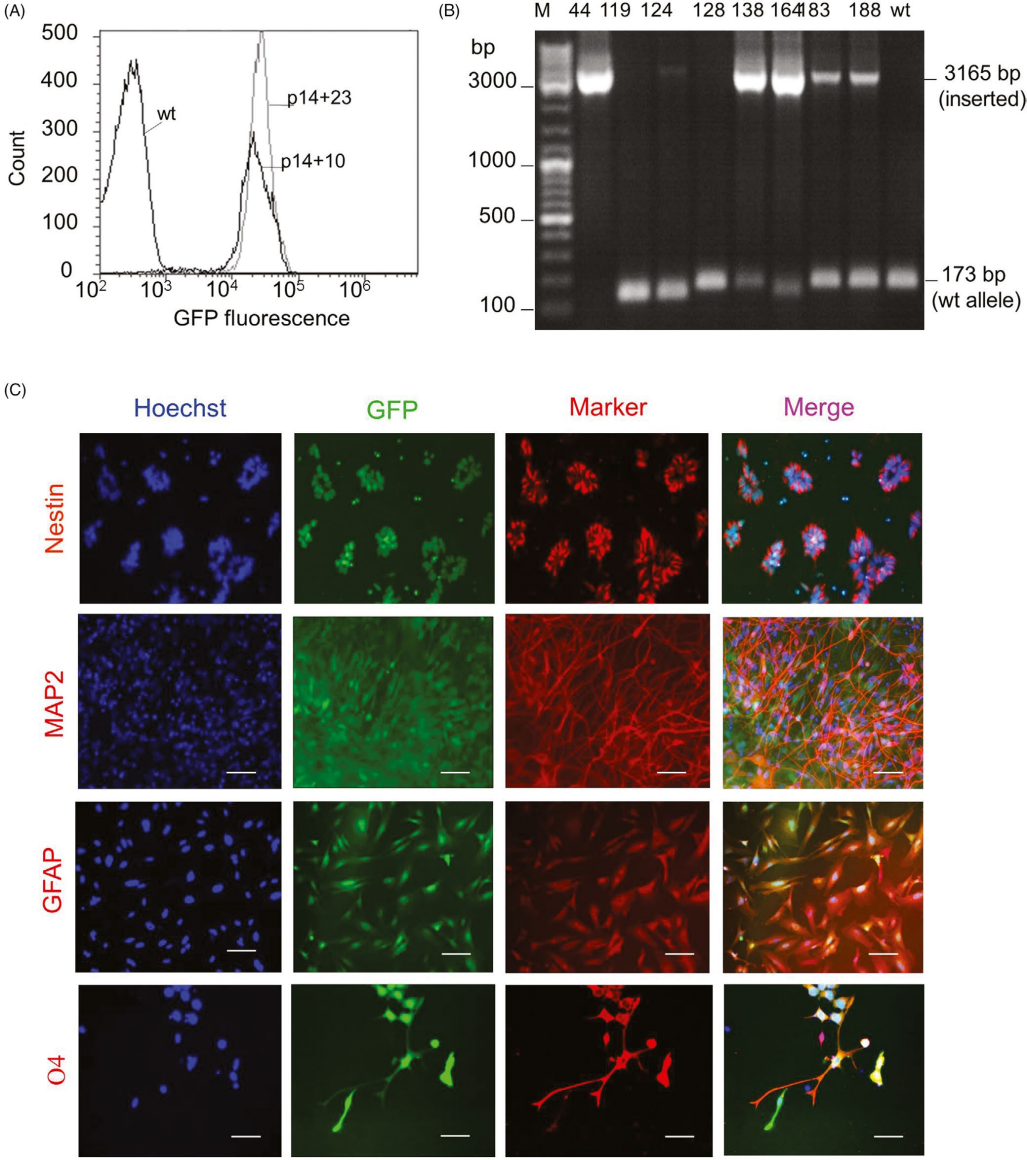
Generation and characterization of genetically modified hiPSC‐NSCs using zinc‐finger nuclease (ZFN) technology. A, Stable expression of eGFP in ZFN‐modified hiPSC‐NSCs during 13 passages of expansion (from p14 + 10 to p14 + 23) in the absence of puromycin selection. B, Identification of mono‐ and bi‐allelic ZFN‐modified hiPSC‐NSC‐clones by amplification of the genomic DNA (gDNA) with P1 + P2 primers located around the integration site (see Figure S1). Intact AAVS1 locus yielded a PCR product with 173 bp in size while amplicon from the targeted allele has the expected size of 3165 bp. C, Immunostaining of bi‐allelic ZFN‐NSC clone 44 with antibodies against NSC marker Nestin. Differentiation of ZFN‐NSCs to neurons, astrocytes and oligodendrocytes was evaluated by immunocytochemistry using antibodies for microtubule‐associated protein 2 (MAP2), glial acidic fibrillary protein (GFAP) and O4, respectively. The expression of transgenic eGFP was retained in all cell lineages. Nuclei were counterstained with Hoechst 33342 (blue). Scale bars: 100 µm

Next, we sought to examine whether ZFN‐based genome editing would affect the propensity of hiPSC‐NSCs to acquire this or other chromosomal anomalies over prolonged passaging. To this end, the targeting vector pAAVS1‐EPG and mRNAs encoding for a pair of ZFNs that target the genomic integration site of AAVS1 locus were co‐transfected into R‐iPSC4‐NSCs at p14. This resulted in 77% eGFP‐positive cells at day two after transfection (Figure S3). Selection with puromycin yielded a pure population of ZFN‐edited NSCs that stably expressed eGFP over at least 23 passages in culture (Figure 2A). To obtain homogeneous NSC populations for subsequent studies, we generated clonal ZFN‐edited NSC lines by single cell subcloning. After 18 days in culture, single NSCs formed eGFP‐positive colonies in 24 out of 192 wells (12.5%). Of these, eight clonal ZFN‐NSC lines were established and characterized in more detail. The AAVS1 locus was successfully targeted in seven out of eight ZFN‐NSC lines: clone 119 showed no integration at the AAVS1 locus, clone 44 carried the bi‐allelic transgene insertion, while clones 124, 128, 138, 164, 183 and 188 carried mono‐allelic transgene insertions (Figure 2B; detailed description of these and other related results is provided in Supplemental Results and in Figures S4‐S6).

### 3.3 Characterization of gene modified hiPSC‐NSCs

To determine whether genetic modification and subcloning affected hiPSC‐NSCs, we assessed their immunophenotype and differentiation potential. Flow cytometry analysis of the bi‐allelic ZFN‐NSC clone 44 demonstrated that the expression level of polysialylated neuronal cell adhesion molecule (PSA‐NCAM) was comparable to that in parental hiPSC‐NSCs (Figure S7A). These cells also retained the ability to form secondary neurospheres (Figure S7B) which could differentiate to microtubule‐associated protein 2 (MAP2)‐expressing neurons (Figure S7C). Moreover, ZFN‐NSCs maintained in monolayer cultures expressed NSC markers Nestin, Sox1 and Pax6, and differentiated towards MAP2‐ and class III β‐tubulin (TUJ1)‐expressing neurons, glial fibrillary acidic protein (GFAP)‐expressing astrocytes and O4‐expressing oligodendrocytes without losing transgenic eGFP expression (Figure 2C and Figure S7D). In addition, electrophysiological analyses showed that neurons derived from parental iPSC‐NSCs and both polyclonal and clonal ZFN‐NSC lines exhibit comparable functional properties (see Supplemental Results and Figure S8).

### 3.4 Molecular karyotyping of ZFN‐edited hiPSC‐NSCs

**Figure 3:**
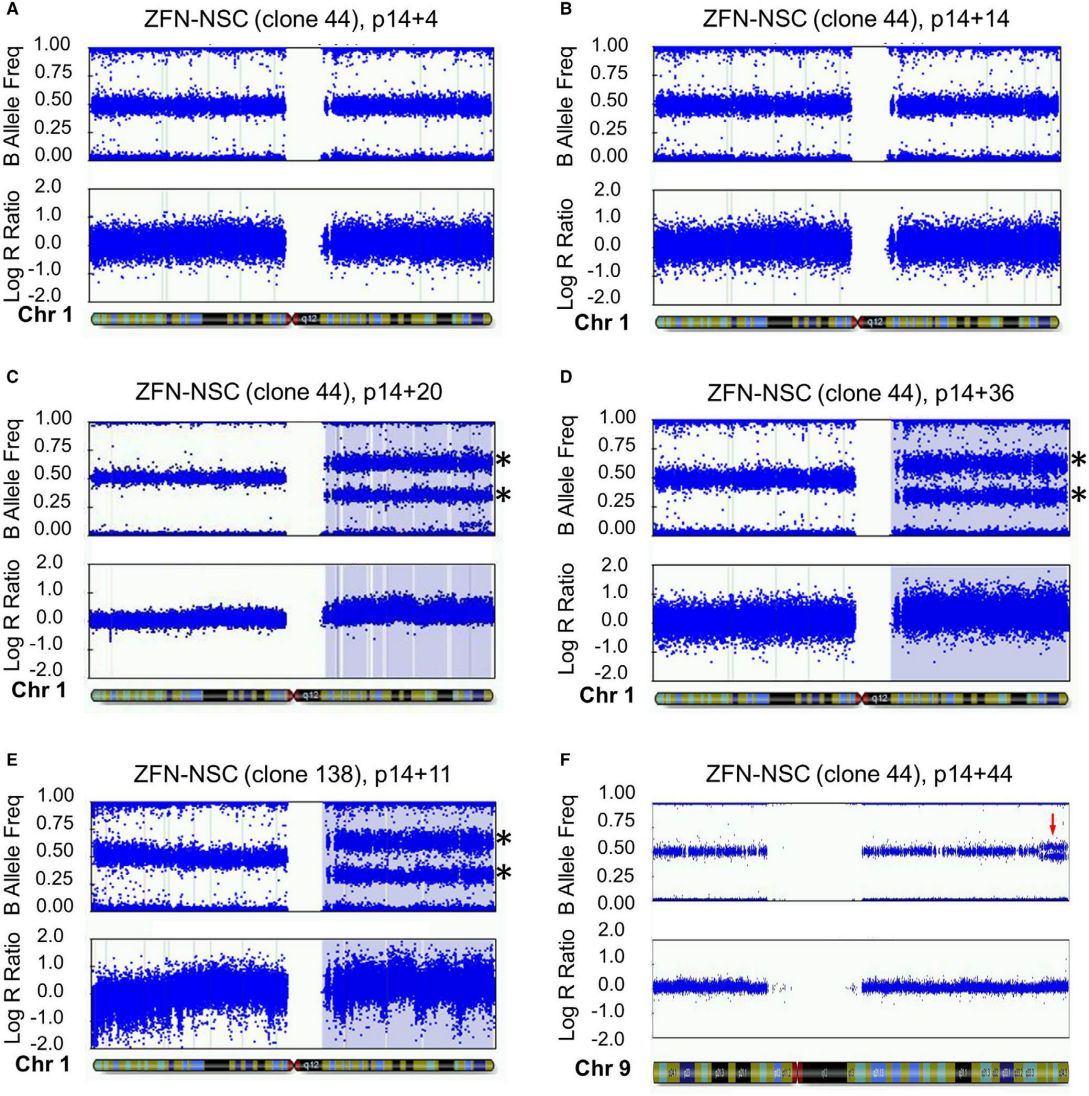
SNP array‐based karyotyping of different preparations of ZFN‐modified hiPSC‐NSCs. B‐allele frequencies (upper panels) and log_2_ R ratios (lower panels) are plotted for chromosome 1 (A‐E) and chromosome 9 (F). The corresponding complete karyotypes are provided in Figures S9‐S14. A, Analysis of the heterogeneous population of ZFN‐NSCs at p4 after genome modification (p14 + 4) did not reveal any major chromosomal abnormalities (see also Figure S9). B, Early after clonal selection at p14 + 14, the bi‐allelic ZFN‐NSC clone 44 did not show any detectable aberrations in Chr1 or any other chromosomes (Figure S10). C‐E, Extended passaging of clonally selected ZFN‐NSCs led to the acquisition of a dup(1)q abnormality (asterisks) as shown for two different batches of clone 44 (batch A at p14 + 20 shown in panel C and Figure S11; batch B at p14 + 36 shown in panel D and Figure S12), and for the mono‐allelic clone 138 at p14 + 11 (panel E and Figure S13). F, ZFN‐NSC expansion for an even longer period led to the acquisition of additional chromosomal abnormalities as exemplified by the duplication of the 10 Mbp telomeric end in the long arm of chromosome 9 observed in clone 44 at p14 + 44 (arrow see also Figure S14)

Next, we sought to determine which chromosomal aberrations occur in hiPSC‐NSCs that underwent the ZFN modification procedure. SNP genotyping of ZFN‐NSCs that were kept in culture for four passages after transfection (p14 + 4) revealed no chromosomal aberrations in these cells (Figure 3A and Figure S9). Chromosomal abnormalities were also not detected after clonal selection of ZFN‐NSCs as shown at p14 + 14 for the clone 44 harbouring bi‐allelic insertion of the transgene cassette (Figure 3B and Figure S10). However, extended passaging of clonal ZFN‐NSCs reproducibly led to the acquisition of a dup(1)q aberration as shown for the bi‐allelic clone 44 at p14 + 20 (Figure 3C and Figure S11) and p14 + 36 (Figure 3D and Figure S12), as well as for the mono‐allelic clone 138 analysed at p14 + 11 (Figure 3E and Figure S13). Cultivation of clonal ZFN‐NSCs for even longer periods of time (p14 + 44) led to the acquisition of additional chromosomal abnormalities, such as duplication of the 10 Mbp large terminal end of the long arm of chromosome 9 (Figure 3F and Figure S14). These results show that dup(1)q is a common aberration both in non‐modified as well as ZFN‐modified hiPSC‐NSCs.

### 3.5 Effect of the dup(1)q on the proliferation rate of ZFN‐NSCs in vitro

**Figure 4:**
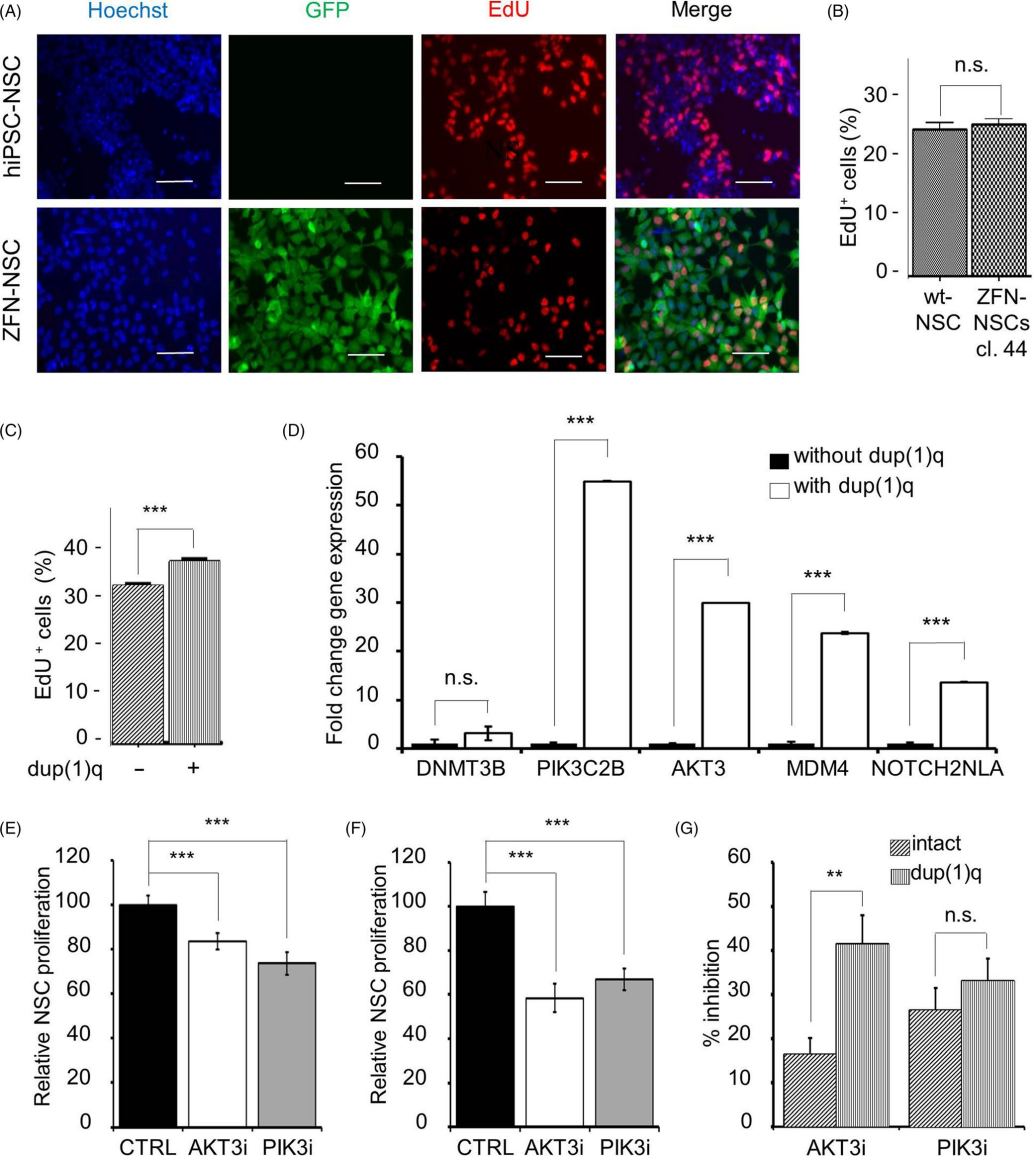
Assessment of the proliferation rate and expression of genes located on chromosome 1q in hiPSC‐NSCs. A, Fluorescence microscopy of EdU‐labelled hiPSC‐NSCs (upper panels) and ZFN‐NSCs (clone 44, lower panels). Cells were incubated with EdU for 2 h and then stained with EdU antibodies to visualize positive nuclei (red). Only transgenic ZFN‐NSCs expressed GFP (green). Nuclei were counterstained with Hoechst 33342 (blue). Scale bars: 100 µm. B, Quantification of the percentage of EdU‐positive NSCs in the experiment shown in panel A based on the scoring of 3087 and 2369 nuclei in non‐modified hiPSC‐NSCs and ZFN‐NSCs (clone 44), respectively (data obtained from two independent experiments, each performed in triplicate). C, The proliferation rate of ZFN‐NSCs (clone 44) with (p14 + 44) and without dup(1)q (p14 + 13) as determined by the EdU incorporation assay. The percentage of EdU‐positive cells was determined in two independent experiments each performed in triplicate. D, RT‐qPCR analysis of *PIK3C2B*, *AKT3*, *MDM4* and *NOTCH2NLA* gene expressions localized on Chr 1q in comparison to *DNMT3B* localized on Chr 20 in ZFN‐NSC clone 44 without (p14 + 11) and with dup(1)q (p14 + 41). E,F, ZFN‐NSCs (clone 44) without (E) and with dup(1)q (F) were cultured for 24 h in the absence (CTRL) and presence of AKT3 inhibitor (AKT3i) MK2206 or PIK3C2B inhibitor (PIK3i) NU7441 (both at 1 μmol/L). The proliferation rate was assessed by the EdU incorporation assay as described for panel A. G, Comparison of the extent of inhibition of EdU incorporation into ZFN‐NSCs with and without dup(1)q after treatment with AKT3i and PIK3i (calculated from data shown in panels E and F). Treatment with AKT3i, but not PIK3i, exerted a significantly stronger inhibition on the proliferation rate of ZFN‐NSCs with dup(1)q than on genetically intact ZFN‐NSCs. n.s.: Non‐significant, **P < .01 and ***P < .001

The Chr1q region harbours the genes which are involved in the regulation of cell survival, proliferation and differentiation, such as *AKT3*, *PIK3C2B*, *MDM4* and *NOTCH2NLA*. Therefore, we used the EdU incorporation assay to determine whether dup(1)q affects the proliferation rate of hiPSC‐NSCs. This analysis showed that ZFN‐mediated genetic modification of NSCs does not affect their proliferative activity in comparison to parental hiPSC‐NSCs (Figure 4A,B). However, there was a significantly higher percentage of EdU‐positive ZFN‐NSCs in cells with dup(1)q compared to those without this aberration (*P* < .0001) (Figure 4C), suggesting that dup(1)q increases the proliferation of hiPSC‐NSCs.

Next, we used RT‐qPCR analysis to assess whether ZFN‐NSCs with and without dup(1)q differ in expression of the above‐mentioned genes. These analyses revealed significant upregulation of the *AKT3* (55‐fold), *PIK3C2B* (30‐fold), *MDM4* (24‐fold) and *NOTCH2NLA* (14‐fold) transcripts in NSCs carrying dup(1)q compared to their genetically intact counterparts (n = 3, *P* < .0001) (Figure 4D). In contrast, expression of *DNMT3B*, which is located on Chr20 and served as a negative control for gene dosage, did not significantly differ between these cell lines. These data show that dup(1)q in NSCs leads to perturbations in expression of genes located on Chr1q and suggest that genes, such as *AKT3* or *PIK3C2B,* might be responsible for their increased proliferation rate.

### 3.6 *AKT3* pathway mediates higher proliferation rate of hiPSC‐NSCs with dup(1)q

To determine whether AKT3 or PIK3C2B signalling pathways mediate the higher proliferation rate of ZFN‐NSCs with dup(1)q, we compared the EdU incorporation in ZFN‐NSCs with and without dup(1)q in the absence and presence of small molecule inhibitors of these protein kinases. This analysis was performed with two independent cell batches, each in triplicate, and reproducibly showed that each inhibitor significantly decreased EdU incorporation both in ZFN‐NSCs with and without dup(1)q (Figure 4E,F). However, the AKT3 inhibitor MK2206 reduced the proliferation of NSCs carrying dup(1)q to a significantly greater extent than the proliferation of karyotypically normal cells (Figure 4G). In contrast, the inhibitory effect of PIK3C2B inhibitor NU7441 did not differ significantly between NSCs with and without dup(1)q (Figure 4G), indicating that AKT3 but not the PIK3C2B signalling pathway at least partly mediates the higher proliferation rate of NSCs with dup(1)q as a consequence of the increased gene dosage.

### 3.7 Dup(1)q increases the proliferation rate of ZFN‐NSCs in vivo

**Figure 5:**
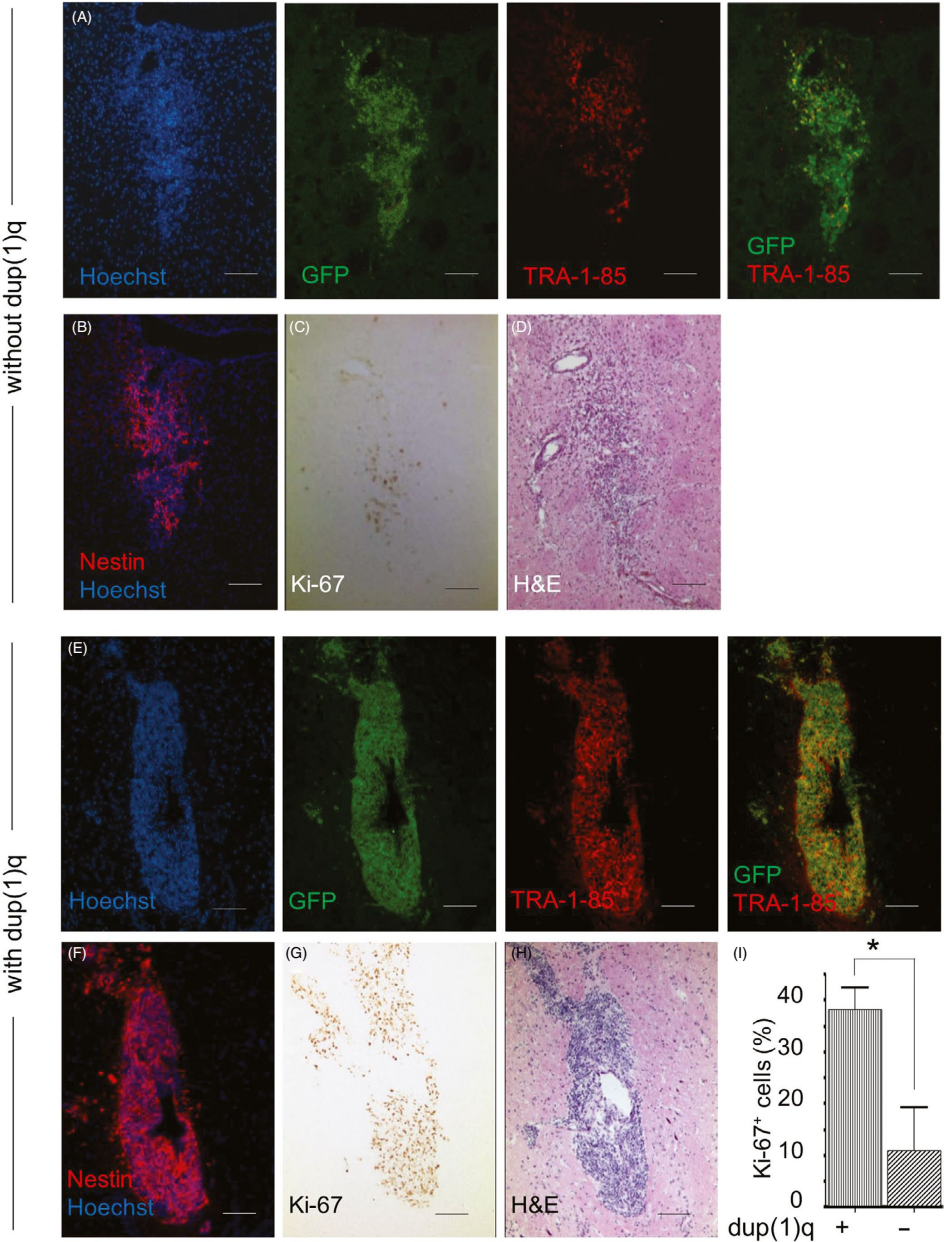
In vivo engraftment and proliferation rate of ZFN‐NSCs with and without dup(1)q. A, E, Representative images of brain slices stained with antibodies against transgenic eGFP and human cell marker TRA‐1‐85 2 weeks after transplantation of ZFN‐NSCs (clone 44) without dup(1)q (A) and with dup(1)q (E) into the striatum of RNU rats. B, F, Expression of Nestin (red) in engrafted ZFN‐NSCs indicates that, independently of karyotype status, most transplanted cells had not differentiated to neural cells. C, G, Mitotic NSCs in the graft area were detected by staining for the proliferation marker Ki‐67 and counterstaining with haematoxylin and eosin (D,H). I, The percentage of Ki‐67‐positive ZFN‐NSCs was significantly higher in engrafted dup(1)q cells than in grafts containing karyotypically normal NSCs. The total number of nuclei counted in animals transplanted with NSCs with and without dup(1)q was 2991 and 452, respectively. Hoechst 33342 was used to label the nuclei (blue). *P < .05. Scale bars: 100 µm

To determine whether the proliferation‐enhancing effect of dup(1)q is also retained in vivo, equal numbers of GFP‐expressing ZFN‐NSCs (clone 44) with or without this chromosomal aberration were transplanted into the striatum of immunodeficient rat brains (n = 3 in each group). Their engraftment and mitotic fractions in the graft area were analysed 2 weeks and 2 months after transplantation. The 2‐week analysis of brain slices revealed that transplanted ZFN‐NSCs in both groups formed well‐delineated grafts which expressed GFP as well as the human‐specific marker TRA‐1‐85 (Figure 5A,E). Immunohistochemistry revealed that most injected cells in both groups expressed this NSC marker Nestin (Figure 5B,F), indicating that at this early time point after transplantation they had not differentiated into neural cell lineages. Assessment of the proliferative activity within the graft area by using an antibody against the proliferation marker Ki‐67 showed that 38 ± 8% of NSCs with dup(1)q were Ki‐67 positive, while only 11 ± 15% of NSCs without duplication expressed this marker (*P* < .05, Figure 5C,D,G‐I).

At 2 months after transplantation, some immature Nestin‐positive ZFN‐NSCs still persisted in the brain (Figure S15A,E) but most ZFN‐NSCs appeared to have differentiated into MAP2‐expressing neurons that overlapped with the human nuclear antigen (HNA) signal in both experimental groups (Figure S15B,F). Confocal microscopy of the section of rat brain transplanted with ZFN‐NSCs carrying dup(1)q revealed that MAP2‐ and HNA‐positive cells projected their neurites into the striatum, which confirmed maturation of ZFN‐NSC‐derived neurons and integration into the brain tissue (Figure S16A,B). Ki‐67 staining revealed that only 0.52 ± 0.52% of NSCs without the duplication and 3.31 ± 2.28% of cells with dup(1)q were mitotically active (Figure S15C,D,G‐I), but this difference was not statistically significant (*P* > .05). These data demonstrate that the pro‐proliferative effect of dup(1)q in hiPSC‐NSCs also persists in vivo and that it is most pronounced in the first few weeks after transplantation before NSCs differentiate to neural cells.

## 4 DISCUSSION

NSCs derived from human ESCs and iPSCs are important vehicles for genetic and molecular therapies in the central nervous system. Genetic modifications of these cells allow for monitoring their differentiation progress,^36^ improve our understanding of neural development and disease,^37^ and may increase their potential for regenerative therapies.^38^ To overcome the disadvantages of genetic modification methods based on random integration, we used ZFN technology for targeted genome modification in hiPSC‐NSCs. Most genome editing studies in the past several years have employed the CRISPR/Cas9 technology because this technology is much more simple, affordable and efficient than ZFN‐ and TALEN‐based systems for targeting any desired single or multiple genomic loci.^14^ However, for introduction of a defined expression cassette into a single genomic safe harbour locus predesigned ZFN‐ or TALEN‐reagents can be equally useful. By using the ZFN‐nuclease commercial kit, we showed that integration of the expression cassette into the AAVS1 locus in hiPSC‐NSCs is highly efficient, enables stable long‐term transgene expression and does not adversely affect the NSC characteristics both before as well as after single cell cloning. This is in agreement with previous studies in ZFN‐modified human foetal NSCs,^31^ TALEN‐modified hiPSC‐NSCs^30^ and CRISPR/Cas9‐targeted human^7^, ^32^ and mouse brain‐derived NSCs.^32^, ^39^ However, the chromosomal integrity and functional consequences that chromosomal aberrations might induce in gene targeted NSCs in vitro and in vivo have not been addressed in these previous studies. Here, we show that prolonged culture of hiPSC‐NSCs leads to the acquisition of dup(1)q independently of whether they were genetically modified by ZFNs or not, and that this aberration increases NSC proliferation in vitro as well as in vivo in the first weeks after transplantation most likely by activation of the AKT3 signalling pathway.

The mechanism responsible for occurrence of dup(1)q in hiPSC‐NSCs is not known. In the literature, several mechanisms have been implicated in the acquisition of genomic instability and aneuploidy in hPSCs. For example, Lamm and coworkers have shown that decreased expression of the transcription factor SRF, which controls the activity of actin cytoskeletal genes, induces replicative stress and chromosomal condensation defects that underlie the ongoing chromosomal instability seen in aneuploid hPSCs.^40^ They suggested that similar mechanism may also operate during initiation of chromosomal instability in diploid hPSCs. In addition, Zhang and coworkers reported that loss‐of‐function mutations in pro‐apoptotic genes or upregulation of anti‐apoptotic genes that may occur in hPSC desensitize them to mitotic stress and enable aneuploid cell survival.^41^ Other studies identified POLD3 and ZSCAN10 as factors involved in maintenance of genomic stability in PSCs. POLD3 is a gene encoding for the accessory subunit of DNA polymerase delta 3, and its loss results in replicative stress, DNA repair impairment, micronucleation and aneuploidy in ESCs.^42^ The embryonic stem cell‐specific transcription factor ZSCAN10 has been shown to protect hPSCs from accumulation of chromosomal structural abnormalities, and defects in apoptosis and in the DNA damage response.^43^ The mechanism which is specifically responsible for the acquisition of dup(1)q in hiPSC‐NSCs will be explored in future studies.

Gains of chromosome 1 have been detected by other groups both in NSCs^25^, ^44^ as well as in human ESCs and iPSCs.^22^, ^23^, ^27^, ^45^ Among them were whole chromosome 1 duplications (trisomy) or unbalanced translocations and interstitial duplications of different segments in its long arm. For example, Weissbein and coworkers observed duplication of the whole chromosome 1q in human PSC‐NSCs but they did not assess its functional consequences.^25^ In contrast, Varela and coworkers detected amplification of a segment of 1q and its translocation onto the telomeric ends of chromosomes 5p, 8q and 13q in long‐term cultured hESC‐NSCs.^44^ They also showed that neuronal differentiation of two aberrant NSC lines was decreased in vitro but this was not systematically observed in all lines that were tested. In addition, ESC‐NSCs carrying the unbalanced 1q translocation failed to integrate into the striatum of the rat brain at 7 weeks after transplantation. Although the study by Varela et al suggested that the duplication of a 1q segment or its translocation onto different recipient chromosomes could hamper the NSC differentiation in vitro and survival in vivo, this effect was not observed in our hiPSC‐NSCs with dup(1)q. The most likely reason for this is in the different nature of duplication identified in our studies.

It is worth noting that the human chromosome 1q corresponds to mouse chromosomes 1 and 3. Interestingly, gain of the entire chromosome 1 was observed in long‐term cultured NSCs derived from mouse ESCs or adult and foetal mouse brain.^46^, ^47^ In the study with mouse foetal brain‐derived NSCs, cells carrying trisomy 1 exhibited increased proliferation and decreased neural differentiation capacity in vitro.^47^ Aberrations in 1q are also one of the most common abnormalities reported among human neoplasms, including haematologic malignancies^48^, ^49^, ^50^ and paediatric brain tumours,^51^, ^52^, ^53^ suggesting that they might be associated with advantages in cell proliferation and survival. Indeed, the proliferation rate of hiPSC‐NSCs carrying dup(1)q in our study was higher than that of karyotypically normal NSCs both in vitro and in vivo. The identification of specific driver gene(s) on chromosome 1q responsible for this effect in NSCs or cancer cells is difficult because more than one gene could be involved in conveying the growth advantage to aneuploid cells. However, number of genes located on chromosome 1q, such as *AKT3*, *PIK3C2B*, *MDM4* and *NOTCH2NL,* are known to be associated with the control of cell proliferation, survival, migration, stress response, oncogenic transformation, neuronal differentiation and intracellular protein trafficking.^54^, ^55^, ^56^, ^57^ Interestingly, these genes were found to be overexpressed in ZFN‐NSCs with dup(1)q and inhibitor studies suggested that *AKT3* pathway may be at least partially responsible for their increased proliferation rate.

Contrary to the observation by Varela and coworkers that hESC‐NSCs carrying chromosomal 1q duplication exhibit altered in vitro differentiation and in vivo engraftment,^44^ the dup(1)q aberration in our study did not affect the integration of ZFN‐NSCs into the rat striatum and 2 weeks after transplantation they still showed enhanced proliferation compared to NSCs without duplication. At this time point, most transplanted cells expressed Nestin and did not exhibit any detectable neuronal cell differentiation, which explains the high proliferation rate observed at this time point. After 2 months, the cells with duplication also exhibited more than a three‐fold higher fraction of dividing cells than control cells. However, the overall number of Ki‐67‐positive cells was very low and not significantly different between control and mutant cell populations. Although we could still detect Nestin‐positive ZFN‐NSCs in the graft area at this time point, many transplanted NSCs differentiated into neurons which appeared to make synaptic connections to neighbouring areas, indicating their functional integration into the host tissue. We observed no tumour formation after 2 months of transplantation, but we cannot exclude the tumorigenicity of cells carrying duplication after a longer period. This should be explored in future studies because this adverse effect occurred 4 years after transplantation of foetal NSCs in a patient with ataxia telangiectasia.^58^

In conclusion, we show that an isolated duplication of chromosome 1q occurs in unmodified and ZFN‐modified hiPSC‐NSCs after prolonged passaging, that this aberration increases NSC proliferation rate in vitro, and that these changes still persist in transplanted cells in vivo. Our preliminary data suggest that the higher proliferation rate of aberrant NSCs is partly mediated by overexpression of the proliferation promoting gene *AKT3* located in duplicated area, but additional studies are required to elucidate the exact mechanism responsible for this phenomenon. It should be noted that in some prior studies no chromosomal abnormalities were observed in the long‐term cultured hPSC‐NSCs.^59^, ^60^ This indicates that acquisition of such abnormalities in cultured cells is not an inevitable event and that conditions might be selected that prevent their occurrence as it was demonstrated in several previous studies.^43^, ^61^, ^62^, ^63^, ^64^, ^65^ Nevertheless, the genomic integrity of all cell products used for regenerative approaches should be carefully assessed and monitored to ensure that they are safe and therapeutically effective.

## CONFLICT OF INTEREST

None of the authors have a conflict of interest to declare. None of the three authors affiliated with the Royan Institute (NZM, ES and HB) are employed by a government agency that has a primary function other than research and/or education, and they are not submitting this manuscript as an official representative or on behalf of their government. This study was entirely performed at the University of Cologne in Germany and was funded by the German Research Foundation (DFG). Results described in this manuscript were used by Narges Zare Mehrjardi in partial fulfilment of the requirements for her PhD degree at the University of Cologne without involvement of any government agency.

## AUTHOR CONTRIBUTIONS

Investigation, formal analysis, validation and writing of the original draft: NZM; investigation, methodology, formal analysis, validation and supervision: MM, AH and AL; investigation and formal analysis: FFH, ES, JPA, SH and NP; resources: MT, JH, SHH and TFW; resources, writing—review and editing: HB; conceptualization and funding acquisition: EAMN; conceptualization, funding acquisition, project administration, supervision, writing—original draft, review and editing: TŠ. All authors have read and approved the final version of the manuscript.

## Supporting information

Supplementary Material

## Data Availability

The data that support the findings of this study are available from the corresponding author upon reasonable request.
