# Supplementary material for: Acquisition of chromosome 1q duplication in parental and genome‐edited human‐induced pluripotent stem cell‐derived neural stem cells results in their higher proliferation rate in vitro and in vivo
Source: Cell Prolif. 2020 Sep 12;53(10):e12892. doi: 10.1111/cpr.12892 (PMC7574866; doi:10.1111/cpr.12892)
Supplement: Supplementary file 1 — Supplementary Material [file CPR-53-e12892-s001.docx]

# Supplemental Material

**Acquisition of chromosome 1q duplication in parental and genome-edited human induced pluripotent stem cell-derived neural stem cells results in their higher proliferation rate *in vitro* and *in vivo***

*Short title:* dup(1)q increases hiPSC-NSC proliferation

Narges Zare Mehrjardi, Marek Molcanyi, Firouze Fulya Hatay, Marco Timmer, Ebrahim Shahbazi, Justus P. Ackermann, Stefan Herms, Stefanie Heilmann, Thomas F. Wunderlich, Nora Prochnow, Aiden Haghikia, Angelika Lampert, Jürgen Hescheler, Edmund A. M. Neugebauer, Hossein Baharvand, and Tomo Šarić

**Index** Page

Supplemental Materials & Methods 2

Supplemental Results 9

Figure S1 13

Figure S2 14

Figure S3 15

Figure S4 16

Figure S5 17

Figure S6 18

Figure S7 19

Figure S8 20

Figure S9 22

Figure S10 23

Figure S11 24

Figure S12 25

Figure S13 26

Figure S14 27

Figure S15 28

Figure S16 29

Table S1 30

Table S2 31

Table S3 31

Table S4 32

Table S5 32

Supplemental references 33

**Supplemental Materials and Methods**

**Culture of human induced pluripotent stem cells (hiPSCs)**

Human iPSC line used in this study (Royan-1 clone 4, R1-iPSC4, also known as RIi007-A) was generated from dermal fibroblasts by retroviral transduction of the four Yamanaka’s reprogramming factors as described previously.^3^ hiPSCs were maintained on irradiated mouse embryonic fibroblasts (MEFs) isolated from CF1 mouse strain (Charles River, Cologne, Germany) at 5% CO_2_ in medium containing DMEM-F12+Glutamax, 20% knock-out serum replacement, 1% non-essential amino acids, 0.1 mM ß-mercaptoethanol (all from Life Technologies, Frankfurt, Germany) and 20 ng/ml FGF2 (Peprotech, Hamburg, Germany). These cells were then adapted to grow under feeder-free culture conditions on Matrigel (low growth factor Matrigel, BD, Heidelberg, Germany) in mTeSR1 medium (Stem Cell Technologies, Cologne, Germany). The medium change was done every day.

**Differentiation of hiPSCs to neural stem cells (NSCs)**

Embryoid body (EB) formation from R1-iPSC4 cells was initiated by detaching the colonies from Matrigel-coated plates by 2 mg/ml of type IV collagenase (Life Technologies) for 15 min and transferring to a bacterial dish containing mTeSR1 medium for 2 days. Then, EBs were cultured for 4 days in mTeSR1 supplemented with 10 nM of SB431542 (SelleckChem, Munich, Germany) and 5 nM dorsomorphin (Sigma-Aldrich, Schnelldorf, Germany). On day 6, EBs were plated on dishes coated with 0.0016% poly-L-ornithine (Sigma-Aldrich) and 1 μg/ml laminin (Sigma-Aldrich) and cultured for 7-10 days in the NSC differentiation medium containing DMEM/F12, 1% N2 (Life Technologies), 20 μg/ml insulin (Sigma-Aldrich), 1.6 g/L glucose (Sigma-Aldrich), 0.02% B27 (Life Technologies), and 20 ng/ml FGF2 according to Koch and coworkers.^4^ To establish a NSC line, plated EB-derived cells were treated with 0.1 mg/ml dispase (Life Technologies) for 15 min at 37°C to isolate the rosette structures from the surrounding flat cells. Clusters were treated first with 1 mg/ml collagenase IV for 5 min and, after removing the enzyme, loose clusters were dissociated with 0.05% trypsin-EDTA (Life Technologies) for 3 min and plated on poly-L-ornithine/laminin coated 6-well plate in the NSC differentiation medium containing 20 ng/ml epidermal growth factor (EGF; Life Technologies). To generate long-term self-renewing NSCs, they were passaged every 4 days (25,000 cells/cm^2^) in the NSC maintenance medium consisting of DMEM/F12 supplemented with Glutamax, 1x N2 supplement, 0.02% B27 supplement, 1.6 g/L glucose, 20 μg/ml insulin, 20 ng/ml FGF2 and 20 ng/ml EGF.

**Construction of a ZFN donor plasmid**

The composition of the transgenic cassette is depicted in the **Figure S1**. The cassette is composed of the EF1α promoter sequence followed by the puromycin resistance gene (PAC) and GFP gene linked by the 2A self-cleaving peptide sequence and terminated with the poly A (pA) sequence. This construct, referred to as the EPG cassette, was amplified from the pLWblast-pEF1α-Puro-2A-GFP-pA vector that was produced in our lab in the backbone vector of the Gateway system (Life Technologies, Frankfurt, Germany). PCR primers used for amplification are listed in **Table S1**. The amplified EPG cassette was then ligated into multiple cloning site (MCS) of the *Xho*I*-*linearized pZDonor plasmid (from the CompoZr® Targeted Integration Kit–AAVS1, Sigma-Aldrich, Schnelldorf, Germany) using the In-Fusion HD cloning kit (Clontech**,** Laye, France) according to manufacturer’s recommendations**.** The resulting donor vector pAAVS1-EPG was used for transfection of hiPSC-NSCs as described in the Materials and Methods section in the main manuscript. Validation of the proper insertion of the transgene cassette into the ZFN cleavage site in intron 1 of the PPP1R12C gene in the AAVS1 locus on chromosome 19 was performed by sequencing of genomic DNA using primers listed in **Table S2**.

**Clone selection**

In order to prepare more homogeneous populations of ZFN-modified hiPSC-NSCs, the single cell clones were generated by diluting 10,000 ZFN-NSCs in a 2+3 mixture of a 24 h hiPSC-NSC-conditioned medium and fresh NSC maintenance medium supplemented with 10 µM of ROCK inhibitor for the first day of culture. The cells were distributed into a 96-well plate to yield one cell per well, which was verified by microscopy. Single cells were kept in culture until they formed properly sized colonies appropriate for further expansion and banking. During this period the medium was replaced by only fresh NSC maintenance medium. Cryopreservation of hiPSC-NSCs was performed in freezing medium containing 90% FCS, 10% DMSO (Applichem, Darmstadt, Germany) and 1x10^6^ cells. The NSC conditioned medium was prepared by culturing 1x10^6^ hiPSC-NSCs on a 60 mm dish coated with poly-L-ornithine/laminin in NSC maintenance medium. After 24 h, the medium was collected, filtered through a 0.2 µm filter (Sterifix, Hamburg, Germany), and used for a single cell subcloning as described above.

**Identification of mono- and bi-allelic ZFN-NSC clones**

To determine which single cell clones carry mono-allelic and bi-allelic transgene integrations, gDNA was isolated by using a DNeasy Blood and Tissue Kit, (Qiagen, Hilden, Germany) from different clones. PCR was done with the primers P1 and P2 that flanked the integration site in the AAVS1 locus (**Figure S1**). Primer sequences are listed in **Table S2**. The PCR reaction was done with an Amplitag Gold 360 Master Mix (Applied Biosystems, Frankfurt, Germany) under the following conditions: denaturation 95°C, 10 min; denaturation 95°C 30 s, annealing 60°C 30 s, extension 72°C 3 min for 40 cycles, and a final extension at 72°C for 7 min. The expected PCR products in the mono-allelic clones were 173 bp and 3165 bp in length, whereas in the bi-allelic clones only one 3165 bp PCR-product was expected.

**PCR to detect specific integration of the donor vector**

To determine whether the EPG construct was properly integrated into the AAVS1 locus, the PCR reaction was done with gDNA isolated from GFP-positive NSCs after puromycin selection. Primers P3 and P4 were used to verify the proper transgene integration at the 5’ side (PCR product: 2956 bp) and primers P5 and P6 to evaluate the integration at the 3’ side (PCR product: 2025 bp) (**Figure S1**). Primer sequences are provided in the **Table S2**. PCR was performed with the Amplitag Gold Master Mix as described above.

**Southern blot**

In order to confirm the proper targeting of the EPG construct into the AAVS1 locus and to identify any potential off-targeting integration sites in ZFN-NSC clones, 25 µg of gDNA from selected NSC clones was digested with EcoRI (Thermo Scientific**)**, separated on an 0.8% agarose gel, and transferred to a nylon membrane (Amersham Hybond XL, GE Healthcare Buckinghamshire, GB). Southern blot analysis was performed by using a GFP probe located in the inserted cassette. The probe was prepared by PCR using a pAAVS-EPG vector as a template and primers F_g_ and R_g_ (**Figure S1**). The probe was then labeled using α-^32^P-dCTP and the Ladderman Labeling Kit (Takara, Otsu, Japan). The expected size of the genomic *Eco*RI fragment that could be detected by autoradiography with this probe in transgenic ZFN-NSCs, but not wild-type hiPSC-NSCs, was 7798 nt (**Figure S1**).

**Differentiation of NSCs to neurons, astrocytes and oligodendrocytes**

To differentiate NSCs to neuronal cells, 10,000 cells/cm^2^ were plated on poly-L-ornithine/ laminin coated plates in the NSC maintenance medium. Next day, medium was changed to DMEM/F12 : neurobasal medium (1:1; Life Technologies;) containing 1% N2 supplement, 1% B27 supplement, 200 μM ascorbic acid (Wako, Neuss, Germany) and cells were cultured for 2 weeks. Afterwards, the medium was changed to DMEM/F12 : neurobasal medium (1:3) containing 0.5% N2 supplement, 1% B27 supplement, and 10 ng/ml BDNF (brain derived neurotrophic factor, R&D, Wiesbaden-Nordenstadt, Germany) and cells cultured for 1 additional week. Half of the medium was changed every other day. Then, differentiated cells were stained with antibodies against TUJ1 and MAP2 to detect neuronal cells.

To differentiate NSCs to astrocytes, 50,000 cells/cm^2^ were plated on poly-L-ornithine/laminin coated plate and cultured in the NSC maintenance medium. After 24 hours medium was changed to DMEM/F12 containing 10% FBS, 1% minimum essential medium non-essential amino acids (MEM NEAA) and 1% L-glutamin (all from Life Technologies) for 7-10 days. 75% of medium was changed every other day. After one week cells were stained with antibody against GFAP to observe astrocytes.

For differentiation to oligodendrocytes, 50,000 cells/cm^2^ were plated on poly-L-ornithine/ laminin coated plate in the NSC differentiation medium. Next day, half of the medium was changed to oligoprogenitor cell (OPC) medium containing DMEM/F12, 2% B27 supplement, 2 mM L-glutamin, 10 μg/ml insulin, 10 μg/ml putrescine, 63 ng/ml progesterone, 50 ng/ml sodium selenite, 40 ng/ml triiodothyronine (T3), 50 μg/ml holo-transferrin and 20 ng/ml EGF (all from Sigma-Aldrich). After one week, the cells were dissociated by 0.05% trypsin–EDTA, 15,000 cells/cm^2^ were plated on poly-L- ornithine/laminin coated plate and treated with OPC medium for 2 weeks. Medium was changed every other day. After 2 weeks, cells were stained with specific antibody for O4 to demonstrate oligodendrocytes.

**RT-PCR**

Total RNA was isolated from iPSCs, EBs and NSCs by Trizol reagent (Life Technologies) following the manufacturer’s recommendations. RNA concentration was measured with a Nanodrop 1000 (Thermo Scientific, Frankfurt, Germany) and the quality was assessed by agarose gel electrophoresis. cDNA samples were synthesized from 1 μg of total RNA using the SuperScript II First-Strand Synthesis Kit and random hexamers (Life Technologies) for priming. PCR was carried out using Dream Taq Master mix (Thermo Scientific, Frankfurt, Germany) and PCR products were analyzed by agarose gel electrophoresis with a DNA ladder mix (SM0331, Life Technologies) to determine the PCR product sizes. PCR primers are listed in the **Table S3**.

**Quantitative real-time PCR**

# cDNA was synthesized from 1 μg of isolated mRNA from ZFN-NSC clone 44 with and without 1q duplication. For PCR amplification, 2 μl of 1:20 diluted cDNA were amplified using 1x SYBR^®^ Green JumpStart^™^ *Taq* ReadyMix^™^ (Sigma-Aldrich) and 0.3 μM of each primer pair in final volume of 20 μl/well. Amplification was performed in MicroAmp 96-well plates (Applied Biosystems) starting with an initial step for 2.0 min at 50°C, 2 min template denaturation/hot start step at 94°C, followed by 40 cycles (94°C for 15 sec, 60°C for 1 min). Quantitative PCR analysis for each sample was performed in triplicates. β2-microglobulin (B2M) was used as an internal control. Relative gene expression values were obtained by normalizing CT (threshold cycle) values of the target genes in comparison with CT values of the housekeeping gene (B2M) using the ΔCT method.

**Immunocytochemistry and immunohistochemistry**

After differentiation, NSCs were fixed with 4% PFA, permeabilized with 0.5 M ammonium chloride (Applichem, Darmstadt, Germany), 0.25% Triton-X100 (Sigma-Aldrich) in 0.1 M PBS for 15 min, and blocked with 5% albumin fraction V (Applichem) for one hour at room temperature. Samples were then incubated overnight at 4°C with primary antibodies against SOX1, PAX6, Nestin ,TUJ1, MAP2, TRA-1-85, human nuclear antigen (HNA) or GFP (see **Table S4)**. Secondary antibodies were added in the blocking buffer and incubated for 60 min at room temperature (**Table S5**). Nuclei were stained with Hoechst 33342 (Life Technologies). Immunocytochemisty samples were embedded in DABCO (Sigma-Aldrich) and observed using Axiovert 200M (Carl-Zeiss) fluorescence microscope equipped with the image processing software Axiovision 4.5.

For immunohistochemistry of the transplanted rat brain tissues samples, two weeks and two months after NSC transplantation the animals were sacrificed by anesthesia overdose i.p. and transcardially perfused with 4% paraformaldehyde (PFA). The brain was then additionally fixed for 24 hr in 4% PFA followed by incubation in 30% sucrose at 4°C and freezing in Tissue-Tek. Cryosections were prepared on a Leica CM3050S cryotome. Stained tissues slices were analyzed under a fluorescence microscope Leica DMRB (Wetzbar, Germany) or Olympus BX71.

**Flow cytometry**

To determine the stability of GFP expression in polyclonal ZFN-NSCs as well as bi-allelic (#44) and mono-allelic (#138) clones of ZFN-NSCs, cells were kept in culture in NSC differentiation medium in the presence of FGF2 and EGF without puromycin for 12 or 7 passages. Cells were passaged every 4-5 days. NSCs were dissociated with 0.05% trypsin-EDTA and analyzed on an Attune flow cytometer (Thermo Fisher Scientific, Darmstadt, Germany) to determine the expression of GFP. In separate experiments 0.5X10^6^ cells were stained for 30 min with primary antibody against PSA-NCAM (Chemicon, clone 2-2B) diluted at 1:100 in PBS including 1% FBS. After washing, cells were incubated with the secondary antibody anti-mouse IgG conjugated to Alexa Fluor 555 (1:100) for 30 min at 4°C and analyzed as specified above.

**Electrophysiology**

Patch clamp experiments were performed with an EPC-10USB amplifier (HEKA electronics, Lambrecht, Germany) and glass electrodes with tip resistances of 2.0-3.5 MΩ, manufactured with a DMZ puller (Zeitz Instruments, Germany). Pipette potential was zeroed prior to seal formation. The external solution contained (in mM): 125 NaCl, 2.5 KCl, 2 CaCl 2, 1 MgCl2, 25 NaHCO3, 1.25 NaH2PO4, and 25 D-glucose, bubbled with carbogen. The internal solution (in mM): 4 NaCl, 135 K-gluconate, 3 MgCl2, 5 EGTA, 5 HEPES, 2 Na2-ATP, and 0.3 Na3-GTP (pH 7.25). All whole-cell recordings were performed at room temperature. For current clamp recordings, cells were clamped to a holding potential of -70 mV by injecting small currents. Voltage clamp experiments were performed directly following current clamp recordings on the same cells. Capacitive transients were compensated for using the PatchMaster software (PatchMaster, HEKA) and the series resistance was compensated by ~20-50%. Voltage ramps (410 ms) from a holding potential of -90 mV to +90 mV were applied and current responses were recorded. Mean current density of voltage-gated sodium channels was calculated by dividing the maximum inward currents by the respective cell capacity derived from C-slow compensation. For mean current density of voltage-gated potassium channels the respective maximum outward current was used.

**Supplemental Results**

**Generation and validation of clonal ZFN-modified hiPSC-NSCs**

Clonal ZFN-edited hiPSC-NSC lines were generated by single cell subcloning. Twenty four colonies were formed after 18 days in culture out of 192 wells containing single NSCs. Eight clones that stably expressed eGFP (**Figure S4A, B**) were randomly selected. PCR amplification of genomic DNA (gDNA) isolated from these clones by using primer pairs P3+P4 and P5+P6 (see the scheme in **Figure S1**) yielded, respectively, products with expected sizes of 2956 and 2025 bp in all clones except clone 119 (**Figure S4C**), indicating that in the most clones the transgene cassette was correctly integrated into the intended target site of the AAVS1 locus. The absence of 2956 bp and 2025 bp amplicons in clone 119 suggested an insertion outside the AAVS1 site because these cells still expressed eGFP fluorescence (**Figure S4A**) and a transgenic construct seemed to be integrated into their genome, as shown by amplification of a 505 bp PCR product (**Figure S4C**) using the primers P7+R_g_ located in the eGFP sequence (see the scheme in **Figure S1**). This result was also confirmed by Southern blot analysis using a radioactively labelled eGFP probe, which revealed the targeted 7798 bp *Eco*RI DNA fragment in all tested clones but not in clone 119 (**Figure S4D**). Clone 188 also carried the reporter cassette in two additional integration sites at other loci (**Figure S4D**, asterisks). These results demonstrate that ZFN genome editing technology successfully targeted the AAVS1 locus in hiPSC-NSCs in most of the tested clonal sublines.

**Identification of mono- and bi-allelic ZFN-NSC clones**

Genomic PCR with primers P1+P2 located at the 5’ and 3’ side of the ZFN cleavage site in the AAVS1 locus was performed to identify which clonal NSC lines carry bi-allelic and mono-allelic integration of the transgenic construct (see the scheme in **Figure S1**). One out of eight tested clones (clone 44) carried the bi-allelic transgene insertion as demonstrated by: a) amplification of the expected 3165 bp PCR product that contained the construct, and b) disappearance of the 173 bp product amplified from the wild type allele (**Figure 2B**). Clone 119 showed no integration at the AAVS1 locus, which corroborated the findings presented above. However, sequencing of gDNA of this clone with the Seq5´F and Seq3´R primers (see the scheme in **Figure S1**) revealed a bi-allelic deletion of 29 nucleotides at the ZFN cleavage site suggesting that the *Fok*I-mediated double-strand break in this clone was repaired by error-prone non-homologous end joining without proper donor vector integration into this locus (**Figure S5A**). Clones 124, 128, 138, 164, 183 and 188 contained mono-allelic insertions as demonstrated by amplification of both the targeted (3165 bp) and the wild-type (173 bp) allele by PCR **(Figure 2B)**. Since clones 124 and 128 showed very weak or no 3165 bp PCR product, we sequenced their gDNA with Seq5´F and Seq5´R primers to clarify the nature of the genetic modifications in these clones. This analysis revealed that the transgene construct was integrated into one allele of the AAVS1 locus of these clones upstream of the ZFN recognition site (**Figure S5B**), which was corroborated by the Southern blot result (**Figure S4D**). In addition, clone 124 carried a deletion of 30 nucleotides at the ZFN-cleavage site in the wild type allele, which agreed with the slightly smaller size of the amplified wild type allele in **Figure 2B**. Sequencing of gDNA of a homozygous clone 44 and a heterozygous clone 138 with Seq5´F and Seq5´R primers (see the scheme in **Figure S1**) also showed deletion of 39 and 41 nucleotides, respectively, at the 5’-end of the integrated construct (**Figure S6A**). However, the sequence of the EPG cassette at the 3’-end of the construct was intact in these clones (**Figure S6B**)**.**

**Functional properties of neurons derived from ZFN-modified NSCs**

In order to investigate the functional integrity of neurons derived from wt- and ZFN-modified iPSC-NSCs, whole cell patch clamp recordings were performed in the voltage and current clamp mode in vi*tro*. We first compared functional properties of neurons derived from wt-iPSC-NSCs and polyclonal ZFN-modified iPSC-NSCs that were used for generation of clonal sublines. All 20 cells investigated in these experiments were characterized by pyramidal somata and multipolar dendritic morphology, as represented by the wt-iPSC-NSC-derived neuron in **Figure S8A**. Depolarizing current injections induced irregular fast adapting action potential (AP) firing in both the wt- and ZFN-modified neurons (**Figure S8B**), as also observed in acute native brain slices of animal models.^1^ An increase of the current amplitude did not increase the firing rate. The firing rates of wt-iPSC-NSC- (n = 8) and ZFN-NSC-derived (n = 12) neurons revealed a clear maximum in response to intermediate depolarizing current injections (**Figure S8B, c**) and decreased upon further depolarization (**Figure S8B, b, a**).

In the voltage clamp mode, depolarizing holding potential depolarization were executed via differentially timed holding potential protocols ranging from -80 to +50 mV in accordance to Gruendken et al.^2^ (**Figure S8C** and **D**, insets). The original current traces of a wt-iPSC-derived neuron in **Figure S8C** exemplarily depicts the stepwise holding potential depolarization from -60 to -30 mV (**Figure S8C**, lower traces) and from -60 to -20 mV (**Figure S8C**, top traces) elicited AP-related current responses. These response patterns could be observed in all tested neurons and were irreversibly blocked by bath application of the voltage-gated sodium channel blocker tetrodotoxin (TTX; 50 µM; **Figure S8C**, grey traces). This finding was concomitantly reflected in all examined wt- and ZFN-modified neurons by a distinct reduction of the exponential, voltage dependent portion of the transmembrane current response in the current density to voltage-relations (**Figure S8D**) and furthermore confirmed by significantly decreased total current responses due to depolarizing holding potential pulses from -80 to +50 mV (**Figure S8E**, inset). In the presence of TTX, all neurons in both groups showed significant reductions in membrane current responses (**Figure S8E**).

Further electrophysiological assessment was performed on neurons derived from wt-iPSC-NSCs and clonally derived ZFN-NSCs (homozygous clone 44). In these set of analyses 10 out of 32 (31%) ZFN-NSC-derived and 12 out of 19 (63%) wt-iPSC-NSC-derived neurons were electrically active and showed APs with a mature shape, including quickly rising upstrokes, an overshoot to positive potentials after hyperpolarization. The resting membrane potential was similar between both groups (about -30 mV) (**Figure S8F**). In voltage-clamp mode, we investigated the currents evoked by ramp stimulation. In electrically active cells, the maximal fast inward current was carried by voltage-gated sodium channels (Na_v_), which were activated at negative potentials, as shown in comparable responses observed in measurements with polyclonal ZFN-NSC-derived neurons (data not shown). Na_v_ current density was calculated as maximal inward current divided by cell capacitance (a measure of the cell size) and revealed comparable values for clonal ZFN-NSC- and wt-iPSC-NSC-derived neurons **(Figure S8G**). Voltage-gated potassium currents (K_v_) were recorded as outward currents at positive potentials. Their current densities (**Figure S8H**) as well as their inactivating component (**Figure S8I**), were also comparable for both wt-NSC- and clonal ZFN-NSC-derived neurons. In summary, neurons derived from both polyclonal and clonal ZFN-NSC lines exhibit comparable functional properties to those derived from parental iPSC-NSCs.

**Immunohistological analyses of brain slices two months after NSC transplantation**

Immunohistological analyses of brain slices at two months after transplantation also revealed the presence of GFP-expressing ZFN-NSCs at the injection site in both animal groups **(Figure S15A,E** and **S16A)**. Some transplanted cells still expressed Nestin indicating that few immature NSCs still persisted in the brain at this time point (**Figure S15A,E**). However, transplanted ZFN-NSCs appeared to have differentiated into neurons as indicated by positive staining for MAP2, a neuronal cell marker that overlapped with the human nuclear antigen (HNA) signal in both experimental groups (**Figure S15B,F**). Confocal microscopy of the section of rat brain transplanted with ZFN-NSCs carrying dup(1)q revealed that MAP2- and HNA-positive cells projected their neurites into the striatum, which confirmed maturation and integration of ZFN-NSC-derived neurons into the rat brain (**Figure S16**). No evidence was found for differentiation of transplanted NSCs to astrocytes (data not shown). Ki-67 staining revealed that, in comparison to the short-term engrafted cells, only 0.52±0.52% of long-term engrafted ZFN-NSCs without the duplication and 3.31±2.28% of ZFN-NSCs with dup(1)q were mitotically active (**Figure S15C,D,G-I**) but this difference was not statistically significant (P>0.05).

**
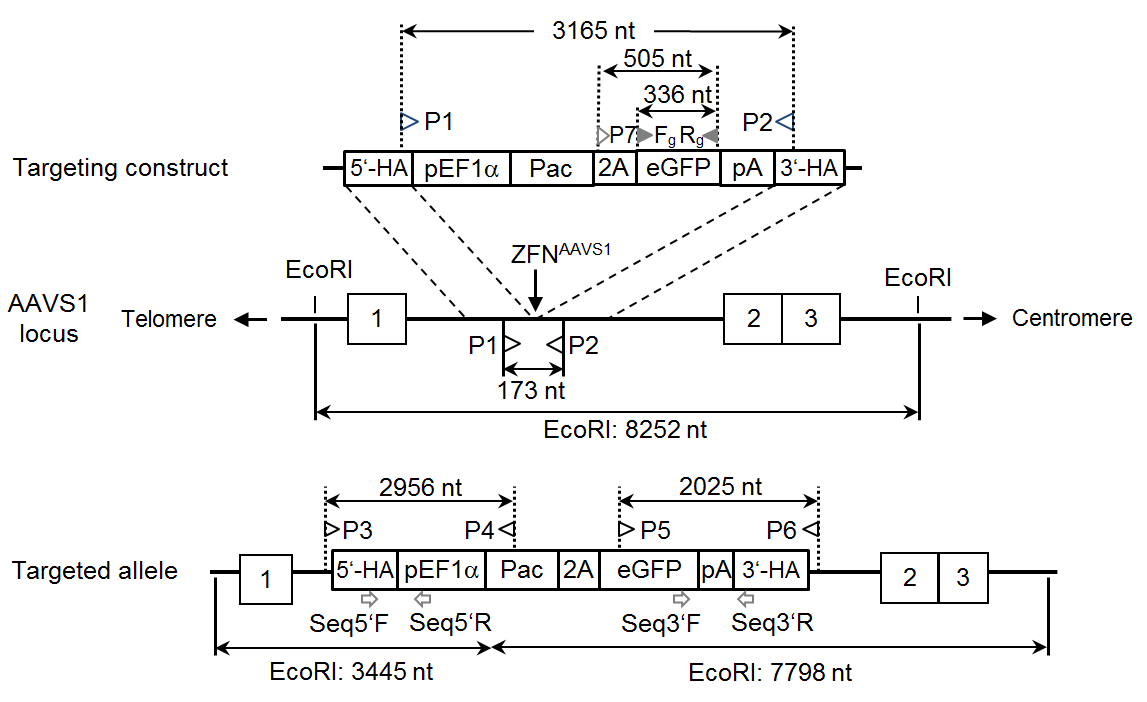
**

**Figure S1. The schematic diagram for targeting the Adeno-Associated Virus Integration Site 1 (*AAVS1*) locus.** Structure of targeting vector, wild type *AAVS1* locus, and ZFN-targeted *AAVS1* locus are shown. The targeting construct pAAVS1-EPG vector (donor vector) was used to insert the transgene cassette into the *AAVS1* locus by homology-directed repair of the double-strand break induced by AAVS1-ZFN-mRNA. The targeting construct contains the EF1-α promoter driving constitutive expression of the eGFP reporter gene and selectable marker for puromycin resistance (Pac) gene flanked by the left (808 nt) and right (795 nt) homology arms (5’-HA and 3’-HA, respectively). Boxes marked 1, 2, and 3 indicate exon regions. The location of *Eco*RI sites up-stream and down-stream of the transgene integration site in the *AAVS1* locus and Southern blot probe (produced by Fg and Rg primers, product size 336 nt, marked by gray arrowheads) located in the integrated cassette are indicated. The sizes of *Eco*RI restriction fragments of the wild-type allele and targeted AAVS1 allele that can be detected with the Southern blot probe are 8252, 3445, and 7798 nt, respectively. P1 and P2 primers located around the integration site are indicated by white arrowheads and yield a PCR product size in the intact chromatid of 173 nt after integration of 3165 nt. P3+P4 (PCR product size 2956 nt) and P5+P6 primer pairs (PCR product size 2025 nt) were used to determine the exact integration at the 5ʹ and 3ʹ ends of the insertion area in the *AAVS1* locus. The P7 and Rg primers located in the integrated cassette (PCR product size 505 nt) were used to determine eGFP expression in different clones. The location of sequencing primers Seq5’F/Seq5’R and Seq3’F/Seq3’R is also indicated.

**
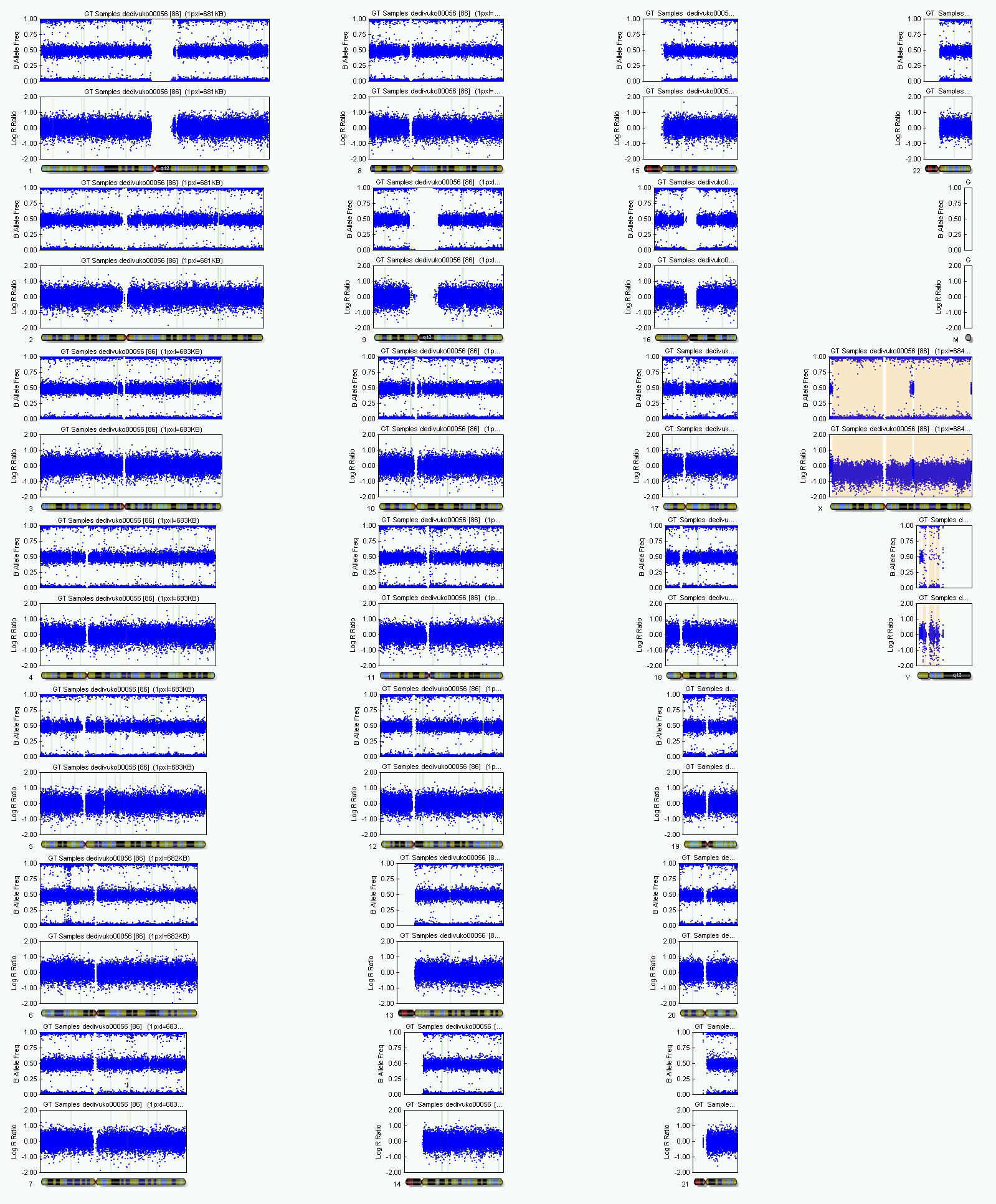
**

**Figure S2. Molecular karyotyping of undifferentiated parental hiPSCs (Ro-iPSC4) at passage 36 (p36) using a whole-genome array-based analysis of single nucleotide polymorphisms (SNP).** B allele frequencies (upper panels) and log_2_ R ratios (lower panels) are plotted for each chromosome for all SNPs on the array located in the corresponding region. Each point is a SNP. This result shows a normal karyotype.


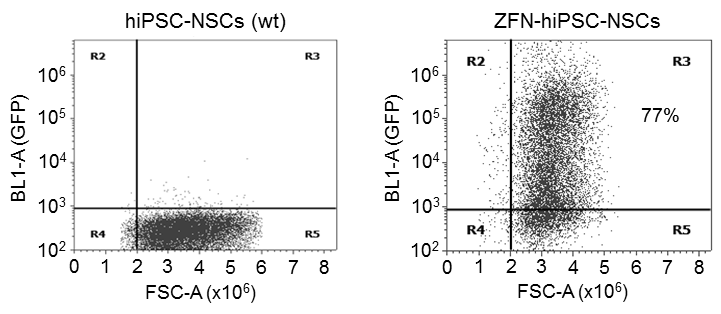


**Figure S3.** Flow cytometry analysis of eGFP expression in hiPSC-NSCs at p14 before (wild type; wt) and 48 h after nucleofection with the pAAVS1-EPG targeting vector.

**
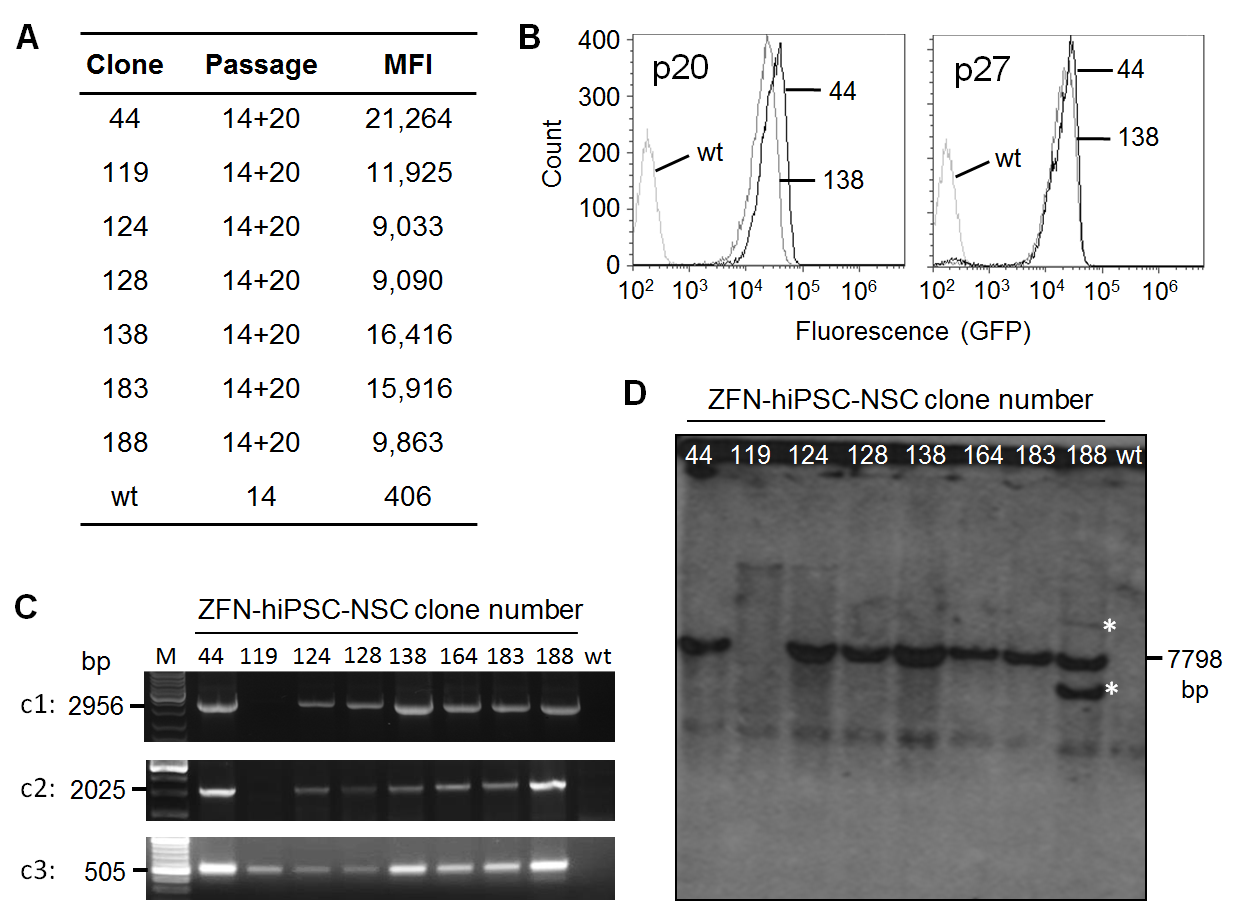
**

**Figure S4. Flow cytometric analysis of GFP expression and assessment of the correct targeting of the transgenic cassette into the AAVS1 locus in selected ZFN-NSC clones.** (**A**) The expression of GFP shown as the mean fluorescent intensity (MFI) in the bi-allelic clone 44, mono-allelic clones 119, 124, 128, 138, 164, 183 and 188, and genetically non-modified wild type (wt) hiPSC-NSCs. Cell passage at which the analyses were performed is indicated. (**B**) Evaluation of GFP expression in a bi-allelic ZFN-NSC clone 44 and mono-allelic clone 138 clone by flow cytometry at p20 (upper panel) and p27 (lower panel) shows stable GFP expression during their *in vitro* expansion. (**C**) Detection of correct integration of the reporter cassette into the AAVS1 locus by genomic PCR with P3+P4 primers (see Figure S1) which amplify a 2956 bp product at the 5’ insertion site (panel c1), and P5+P6 primers which amplify a 2025 bp product at the 3’ insertion site (panel c2). Seven out of eight screened clones harbored the integrated transgene in the proper position. PCR performed with the P7+Rg primers also showed stable integration of the eGFP coding sequence into the genome of ZFN-NSCs as demonstrated by amplification of the expected 505 bp PCR product (panel c3). **(D**) Confirmation of the correct integration of the reporter cassette into the AAVS1 locus by Southern blot analysis. Autoradiogram shown was obtained after hybridization of the *EcoR1*-digested genomic DNA (gDNA) with the GFP probe which was produced by amplification with F_g_+R_g_ primers using the ZFN donor vector as a template (see the scheme in Figure S1). All ZFN-NSC clones analyzed except clone 119 showed the expected 7798 bp band which corresponds to the EcoR1 DNA fragment in the successfully targeted AAVS1 locus. In clone 188 the GFP probe recognized the correct DNA fragment as well as DNA fragments from presumable off-target sites (indicated by asterisks). No specific signal was detected in the gDNA from parental wt-hiPSC-NSCs that served as a negative control in this analysis.

**
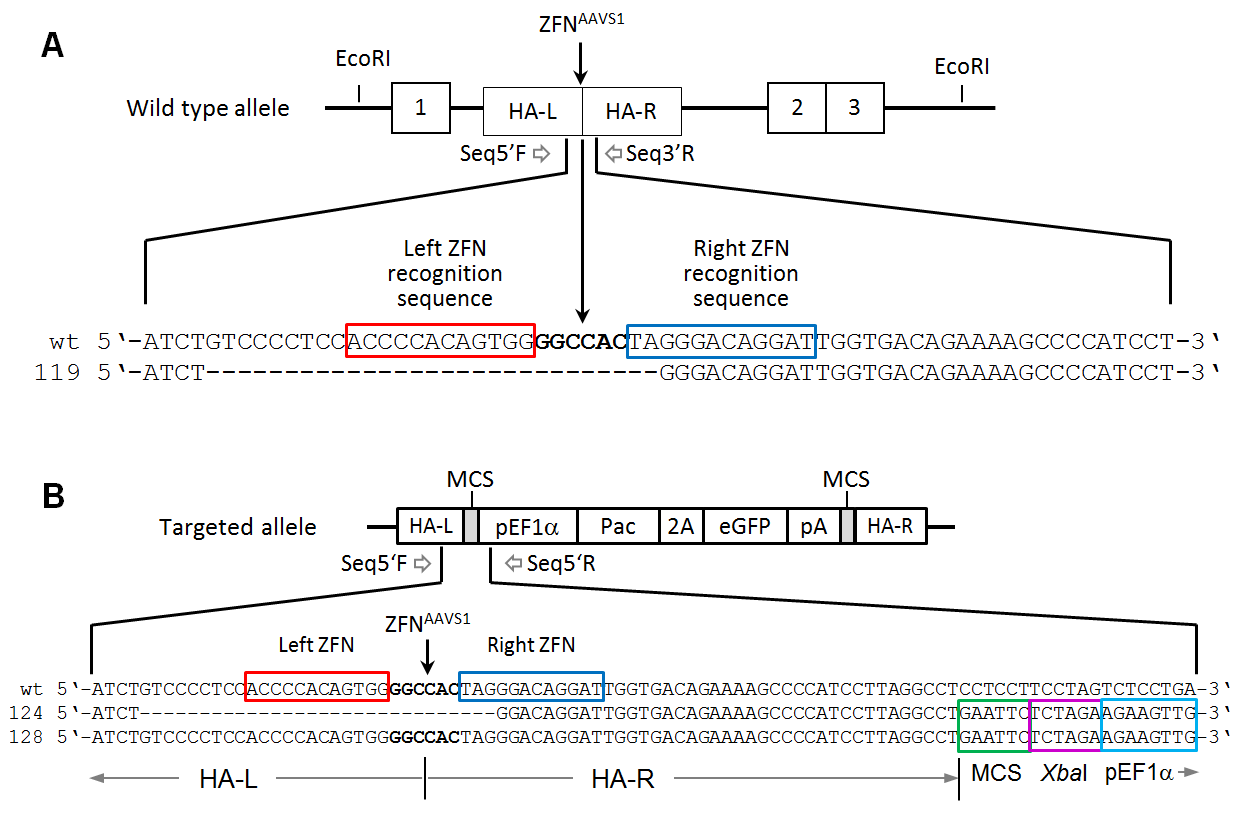
**

**Figure S5. Closer analysis of the AAVS1 integration site in selected ZFN-NSC clones by gDNA sequencing. (A**) Sequencing of a PCR product amplified using primers P3 and P4 (see Figure S1) and gDNA from ZFN-NSC clone 119 with Seq5´F and Seq3´R primers located within the left (HA-L) and the right homology arms (HA-R), respectively. No transgene integration could be detected in this clone at this genomic locus but the ZFN-mediated double-strand break was repaired by non-homologous end joining that resulted in the bi-allelic deletion of 29 nucleotides in this locus. The region between the left (red box) and right ZFN recognition sequence (blue box) is the area over which the *Fok* I restriction endonuclease domains dimerize and cleave the DNA to make a double strand break. (**B**) Sequencing of a PCR product using primers P3 and P4 (see Figure S1) and gDNA from ZFN-NSC clone 124 and 128 with Seq5´F and Seq5´R primers located in the left homology arm (HA-L) and pEF1α vector sequence, respectively (see Figure S1). The integration of the vector cassette was confirmed in both mono-allelic clones but the clone 124 showed deletion of 30 nucleotides at the ZFN-cleavage site in the targeted allele which was absent in the clone 128 and in the wild type (wt) sequence. Sequences in the targeted allele corresponding to the donor vector’s multiple cloning site (MCS) sequence, the *XbaI* recognition site and the beginning of the EF1α promoter sequence are indicated in the corresponding boxed areas.

**
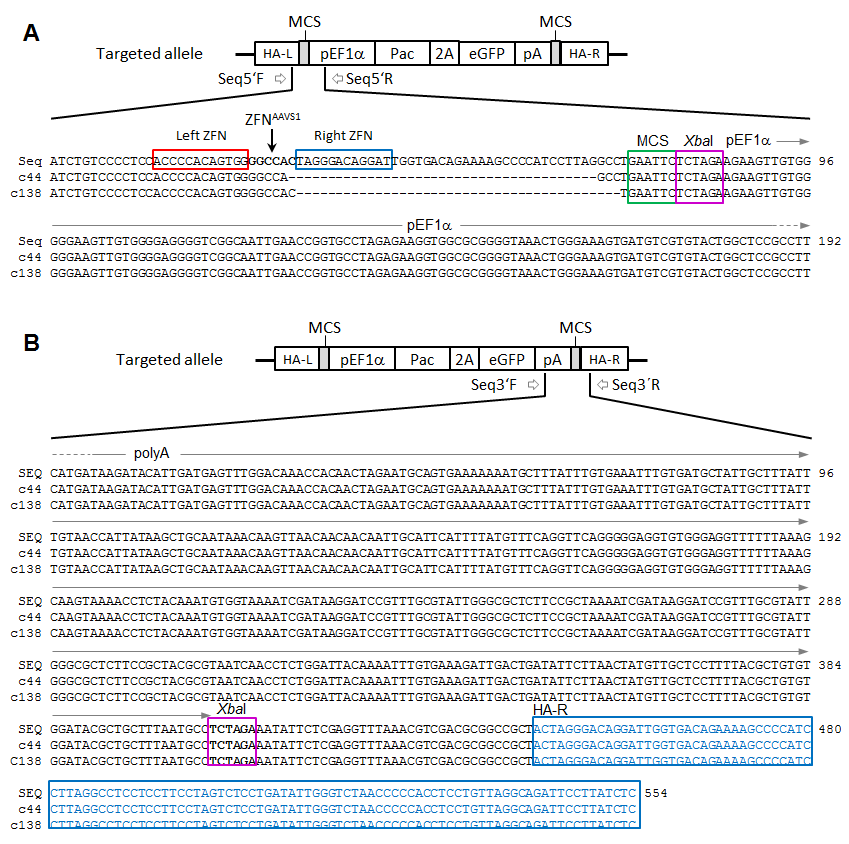
**

**Figure S6. Confirmation of the proper donor cassette integration into the AAVS1 targeting site by Sanger sequencing in mono- and bi-allelic ZFN-NSC clones.** (**A**) Sequencing result of a PCR product obtained with primers P3 and P4 (see Figure S1) and gDNA isolated from bi-allelic ZFN-NSC clone 44 and mono-allelic clone 138. Sequencing was done with Seq5´F and Seq5´R primers (see Figure S1) and the sequences were aligned with the predicted sequence spanning the left homology arm (HA-L) and the immediate 5’ region of the transgene cassette harboring the partial multiple cloning site (MCS) sequence, the *XbaI* recognition site and the initial part of the EF1α promoter sequence. Both clones carry a short deletion in the genomic sequence at the 5’-end of the insertion which did not disrupt the targeting construct. (**B**) Sequencing of PCR product obtained with P5 and P6 primers (see Figure 2A) using as a template the gDNA isolated from ZFN-NSC clones 44 and 138. Sequencing was performed with Seq3´F and Seq3´R primers (see Figure S1) and the obtained sequences showed perfect alignment with the sequence (SEQ) predicted to be found at the 3’ integration side. HA-R: right homologous arm.

**
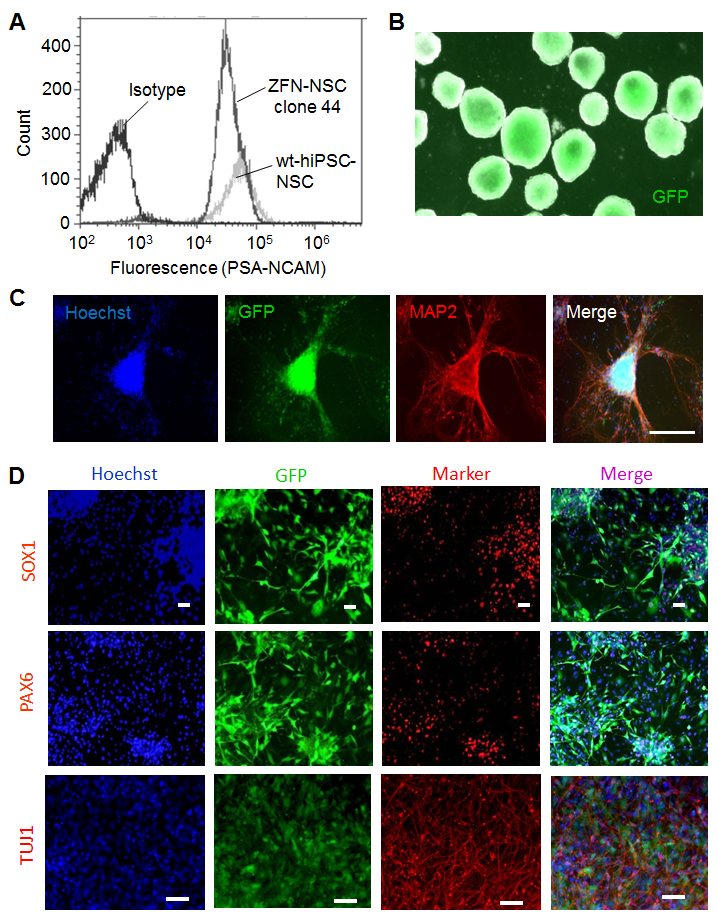
**

**Figure S7. Characterization of the bi-allelic ZFN-NSC clone 44.** (**A**) Comparison of expression of PSA-NCAM in ZFN-NSC and wild-type hiPSC-NSCs by using flow cytometry showed expression of PSA-NCAM in 97% and 95% of cells, respectively. (**B**) Secondary neurospheres were formed from enzymatically dissociated ZFN-NSCs after 2 days in a static culture. The figure is an overlay of bright field and GFP (green) images. (**C**) Differentiation of adherent secondary neurosphere to neurons expressing pan-neuronal marker MAP2 (red) and GFP transgene (green). (**D**) Immunostaining of ZFN-NSC with antibodies against NSC markers SOX1 and PAX6 and staining of neurons derived from these NSCs for pan-neuronal markers TUJ-1. The expression of transgenic eGFP was retained in all cell lineages. Nuclei were counterstained with Hoechst 33342 (blue). Scale bars: 100 µm.

**
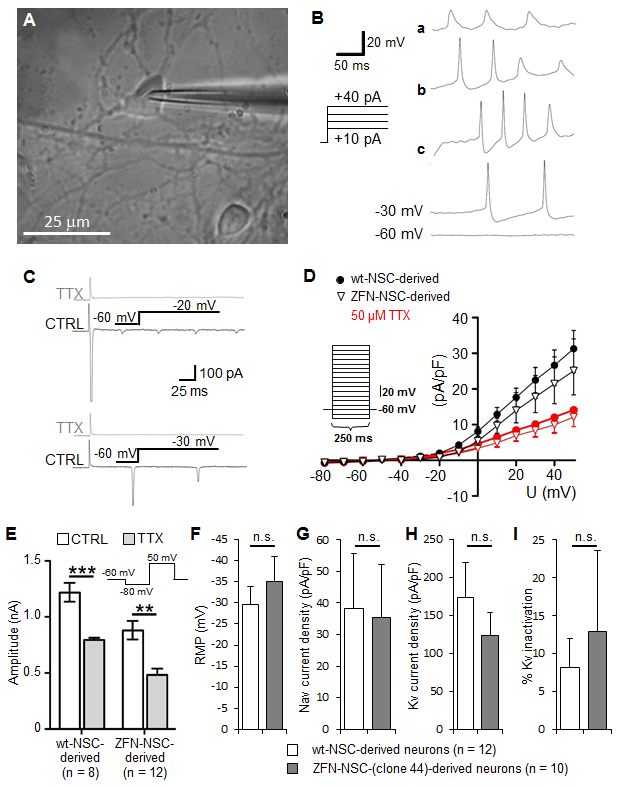
**

**Figure S8. Electrophysiological characteristics of ZFN-NSC-derived neurons.** (**A**) Representative image of a parental wild-type hiPSC-NSC-derived neuron (upper panel) with attached glass pipette for whole cell patch clamp recordings *in vitro*. Image of a ZFN-NSC-derived neuron is shown in a lower panel. (**B-I**) Electrophysiological properties of wt-iPSC-NSC-derived and ZFN-NSC-derived re-differentiated neurons. (**B**) Action potentials (AP) recorded by the whole cell current clamp of the wt-iPSC-NSC derived neuron shown in A. The firing rates were maximal in response to intermediate depolarizing current injections (c) and decreased upon further depolarization (b, a). (**C**) AP related inward currents recorded under stepwise depolarization of the holding potential in the voltage clamp mode (indicated by the insets). These events were irreversibly blocked upon application of the voltage-gated sodium channel blocker tetrodotoxin (TTX) at the concentration of 50 µM (grey traces). (**D**) Reduction in voltage dependent current amplitudes in parental iPSC-NSC- and ZFN-NSC-derived neurons shown as the current density to voltage-relations (pA/pF) in response to a rectangular depolarizing holding potential depolarizations. The effect of 50 µm TTX is also shown (red curves). (**E**) The impact of bath application of 50 µM TTX on the total membrane current amplitude due to hypo- and depolarization from -80 to 50 mV in wt-iPSC-NSC- (n=8) and ZFN-NSC-derived (n=12) neurons. (**F**) Resting membrane potential (RMP) of cells directly after break does not differ between neurons derived from wt-iPSC-NSCs (n=12) and bi-allelic ZFN-NSCs, clone 44 (n=10). Only cells that displayed an AP were analyzed. Sodium current density was determined as maximal inward current (**G**) and potassium current density determined as maximal outward current (**H**) during a voltage ramp divided by cell capacitance as measure for cell size. (**I**) Percent of Kv inactivation is calculated as outward current at the end of the ramp divided by maximal outward current. Prominent inactivation indicates the expression of the delayed rectifier potassium channels. Data in panels E-I are shown as mean ± SD. ** P<0.005, *** P<0.0005. n.s. – not statistically significant.

**
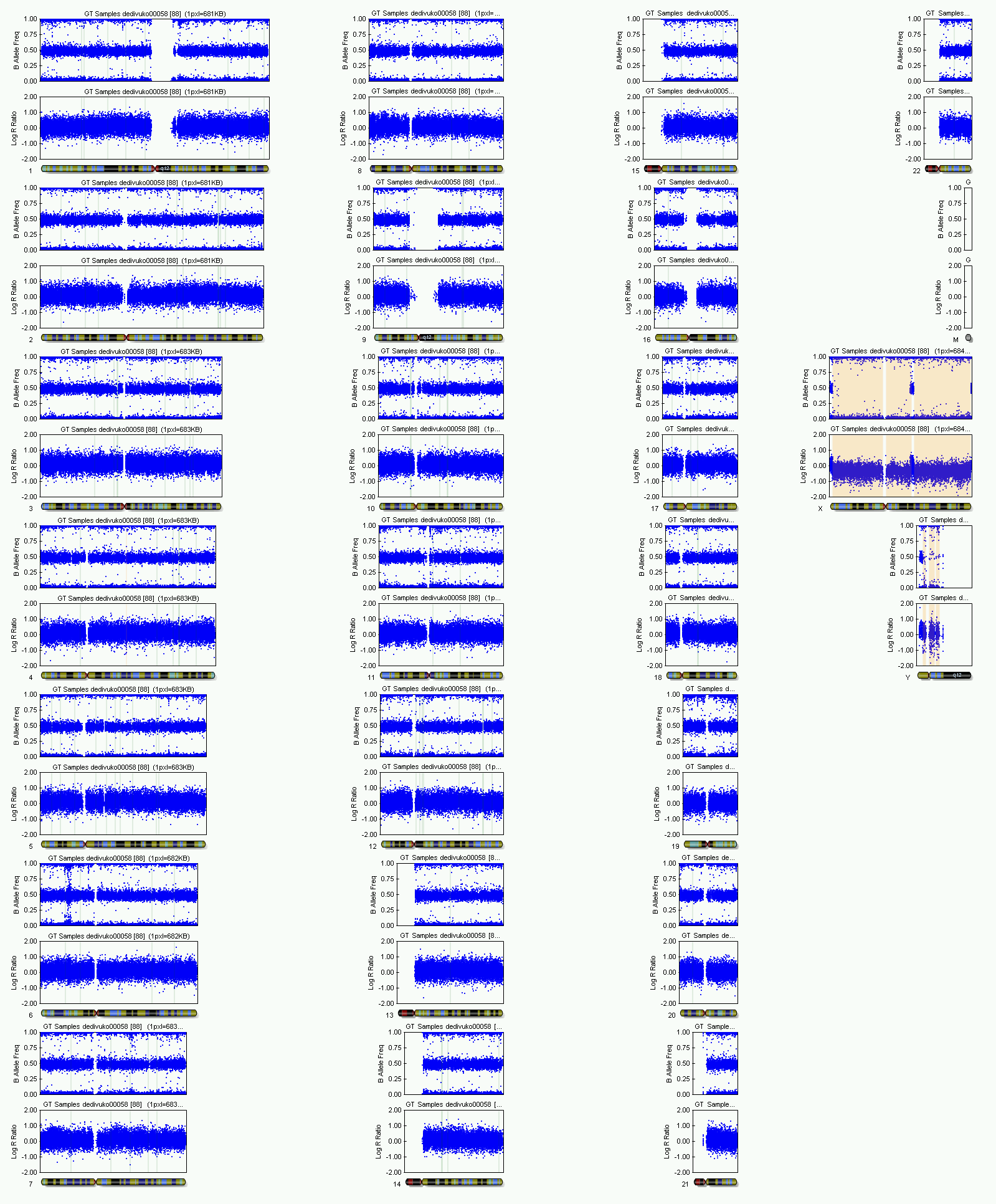
**

**Figure S9. SNP-based molecular karyotyping of a heterogeneous population of ZFN-modified hiPSC-NSCs before clonal selection.** Analysis was performed at passage designated as p14+4, which means that parental hiPSC-NSCs were genetically modified with the ZFN technology at p14 and that ZFN-NSCs were then further expanded for 4 additional passages before karyotype analysis was performed. B allele frequencies (upper panels) and log_2_ R ratios (lower panels) are plotted for each chromosome. Each point is a SNP. No major chromosomal abnormalities could be detected in this analysis.

**
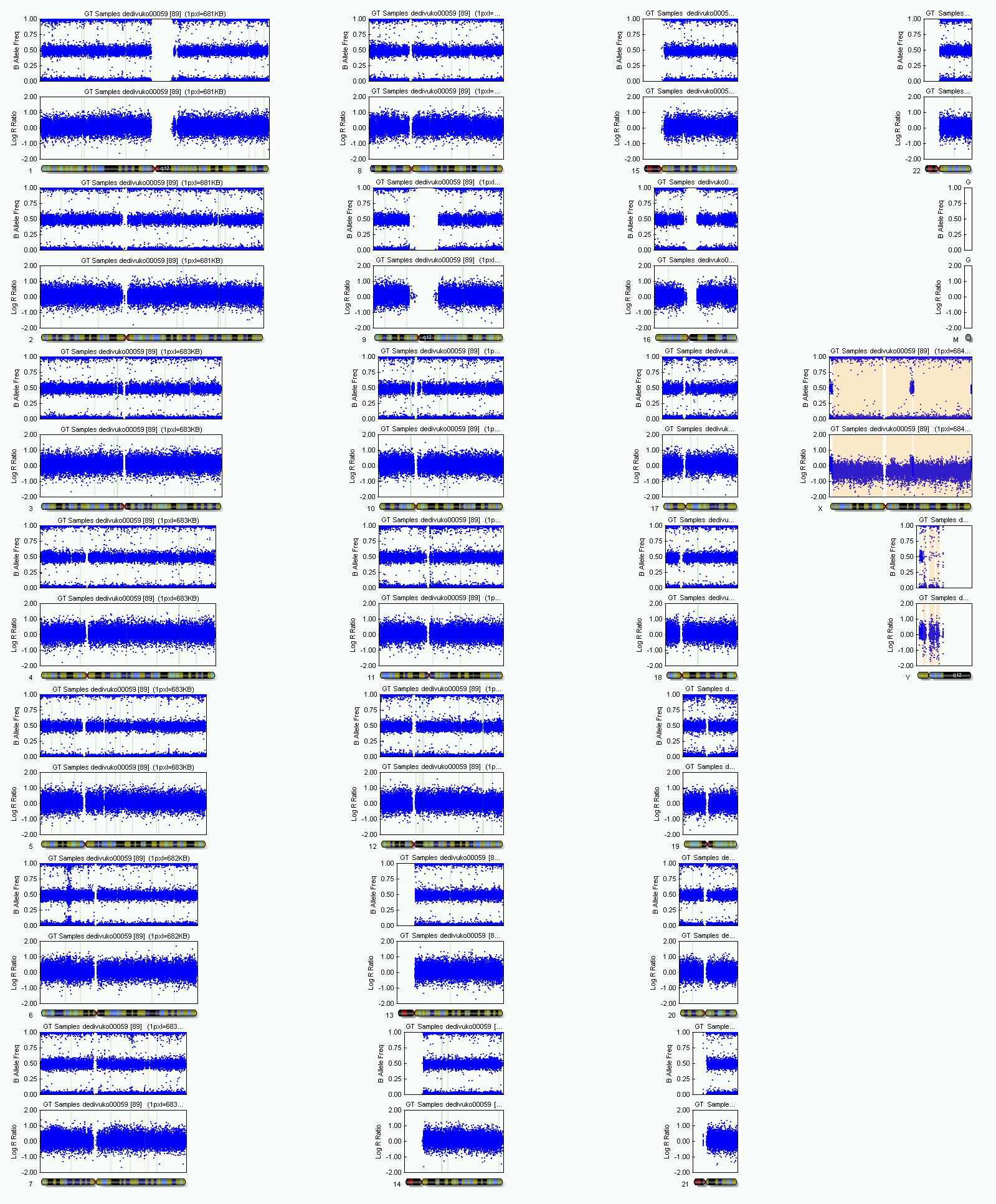
**

**Figure S10. Molecular karyogram of clonally selected ZFN-modified hiPSC-NSCs (bi-allelic clone 44).** The analysis was done at p14 after genetic modification with ZFN (p14+14) using whole-genome SNP-genotyping. B allele frequencies (upper panels) and log_2_ R ratios (lower panels) are plotted for each chromosome. Each point is a SNP. No major chromosomal abnormalities were identified.

**
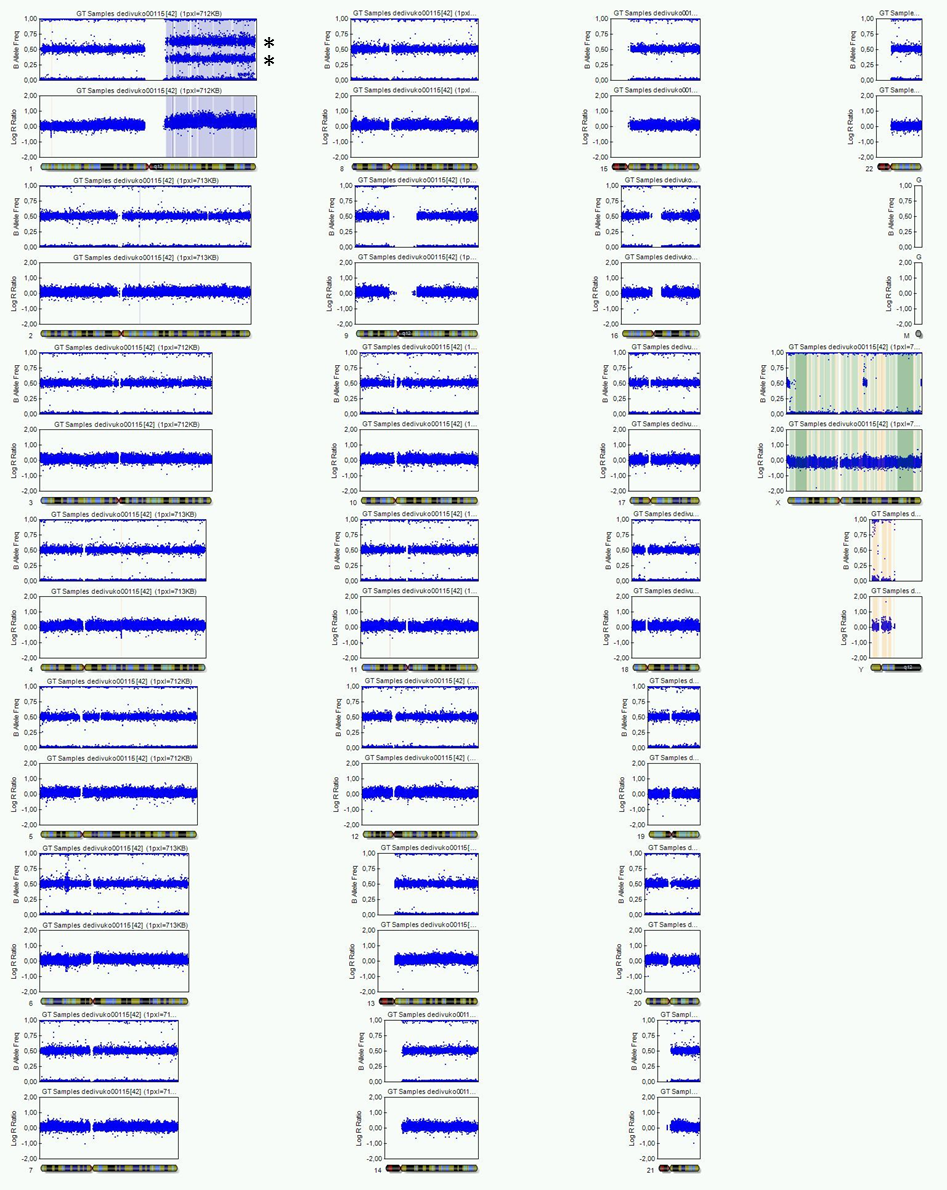
**

**Figure S11. SNP-based genotyping of clonal ZFN-NSC line after prolonged passaging (p14+20).** Results shown were obtained for the bi-allelic ZFN-NSC clone 44 at p14+20 and show the isolated acquisition of dup(1)q in these cells (asterisks). Results are presented as described in the legend for Figure S9.

**
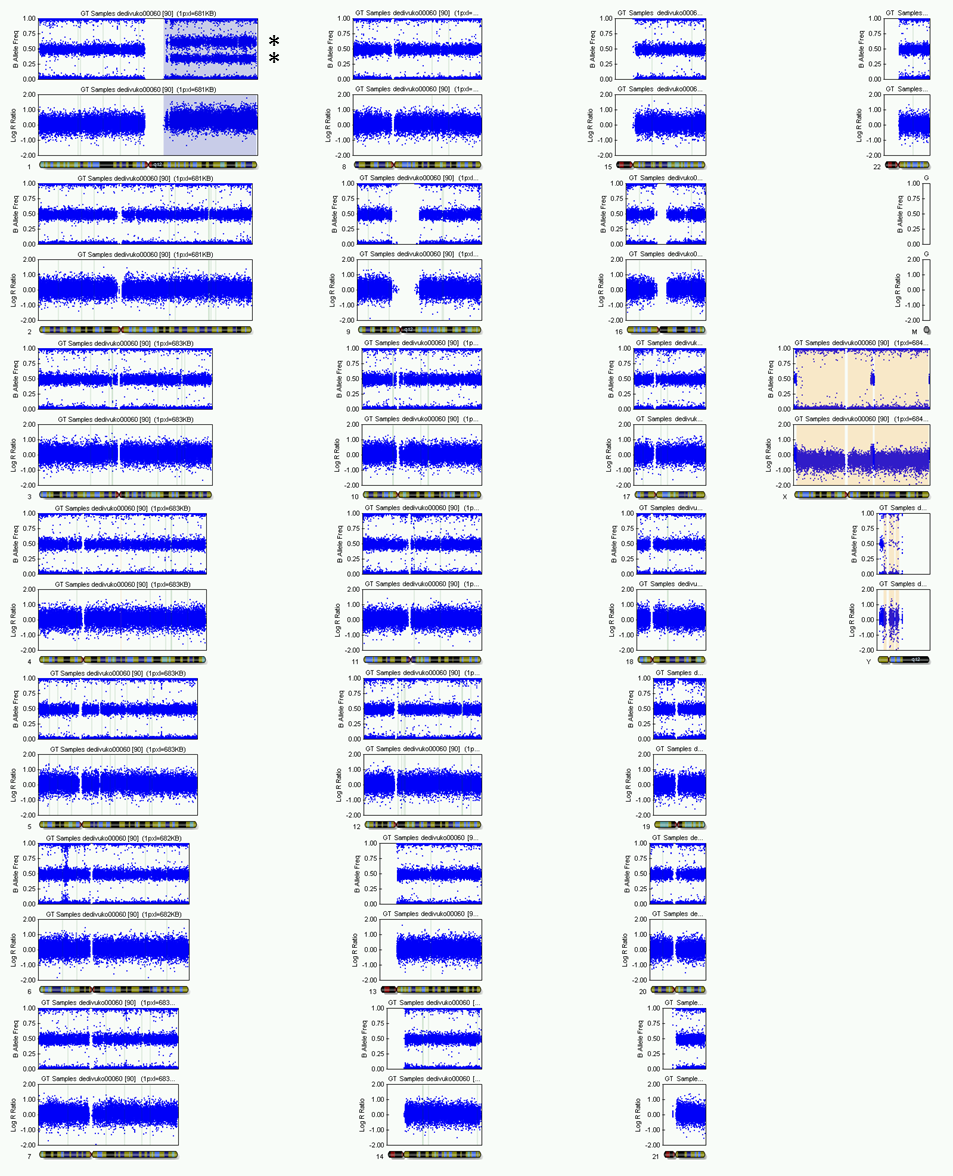
**

**Figure S12. SNP-based genotyping of clonal ZFN-NSC line after prolonged passaging (p14+36).** Results shown were obtained for the bi-allelic ZFN-NSC clone 44 at p14+36 and confirm in an independent experiment the findings presented in Figure S11 about the presence of duplication of the long arm of chromosome 1in these cells (asterisks). No additional chromosomal abnormalities were found.


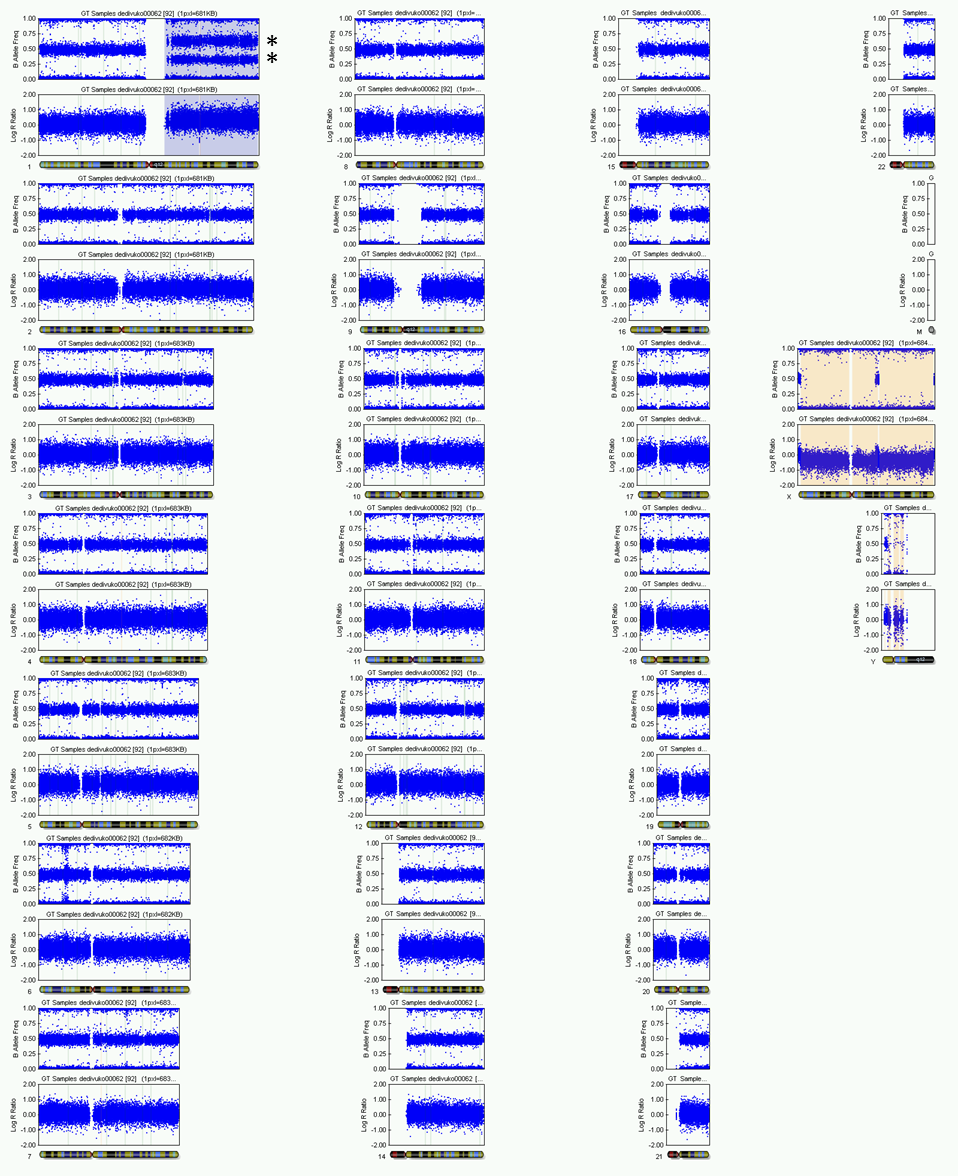


**Figure S13. Molecular karyotyping of mono-allelic ZFN-NSC line (clone 138) at p14+11.** Analysis was performed by using a whole-genome SNP-based array and demonstrates the presence of dup(1)q in these cells (asterisks), confirming the findings shown in Figures S11 and S12 for the bi-allelic ZFN-NSC clonal line 44. Results are presented as described in the legend for Figure S9.

**
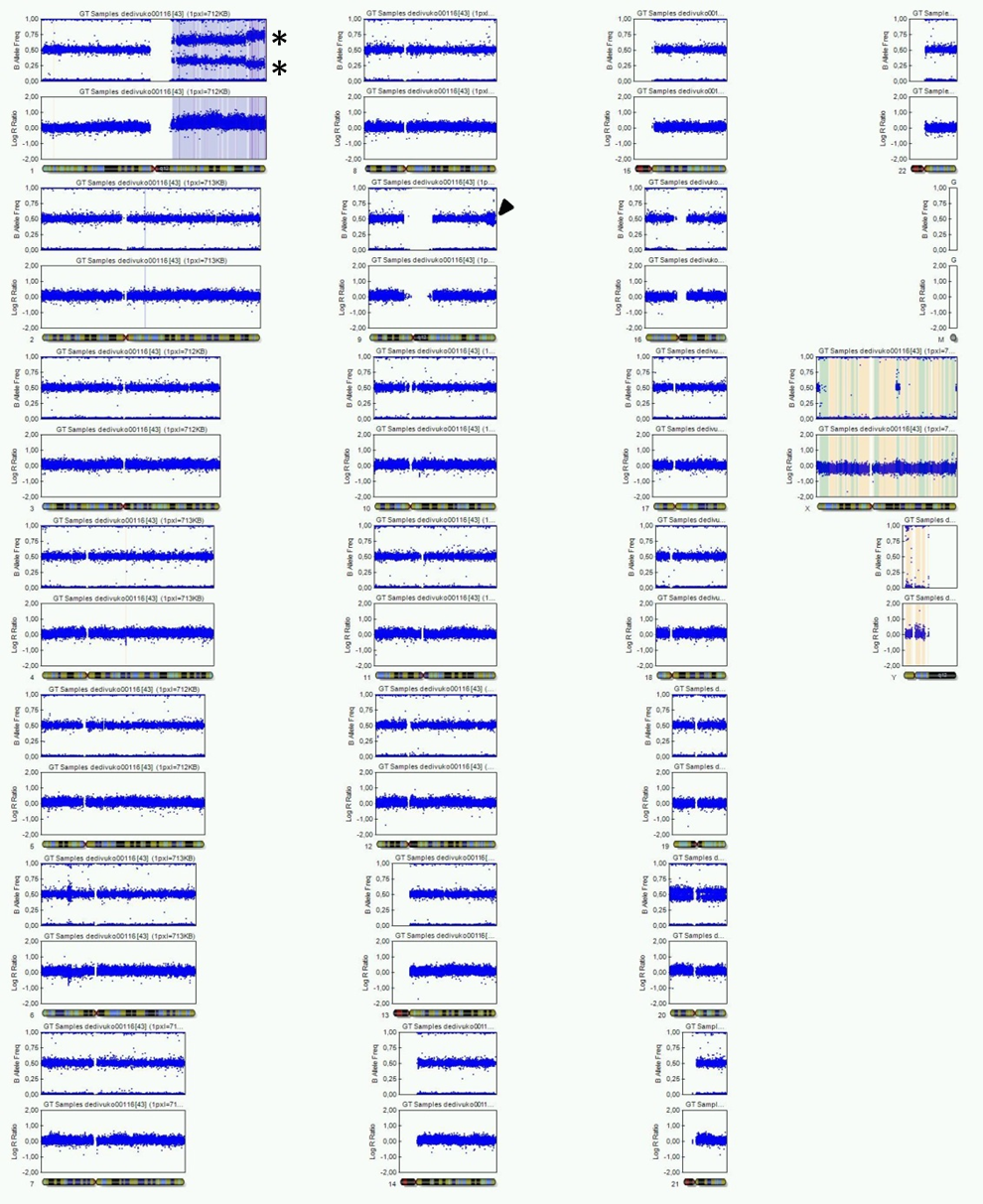
**

**Figure S14. Accumulation of additional chromosomal abnormalities in long-term cultured ZFN-modified hiPSC-NSCs.** Molecular karyogram of the clonal ZFN-NSC line (clone 44) at p14+44 showing duplication of the long arm of chromosome 1 (asterisks) and acquisition of the ~10 Mbp region at the telomeric end in the long arm of chromosome 9 (arrowhead). Results are presented as described in the legend for Figure S9.

**
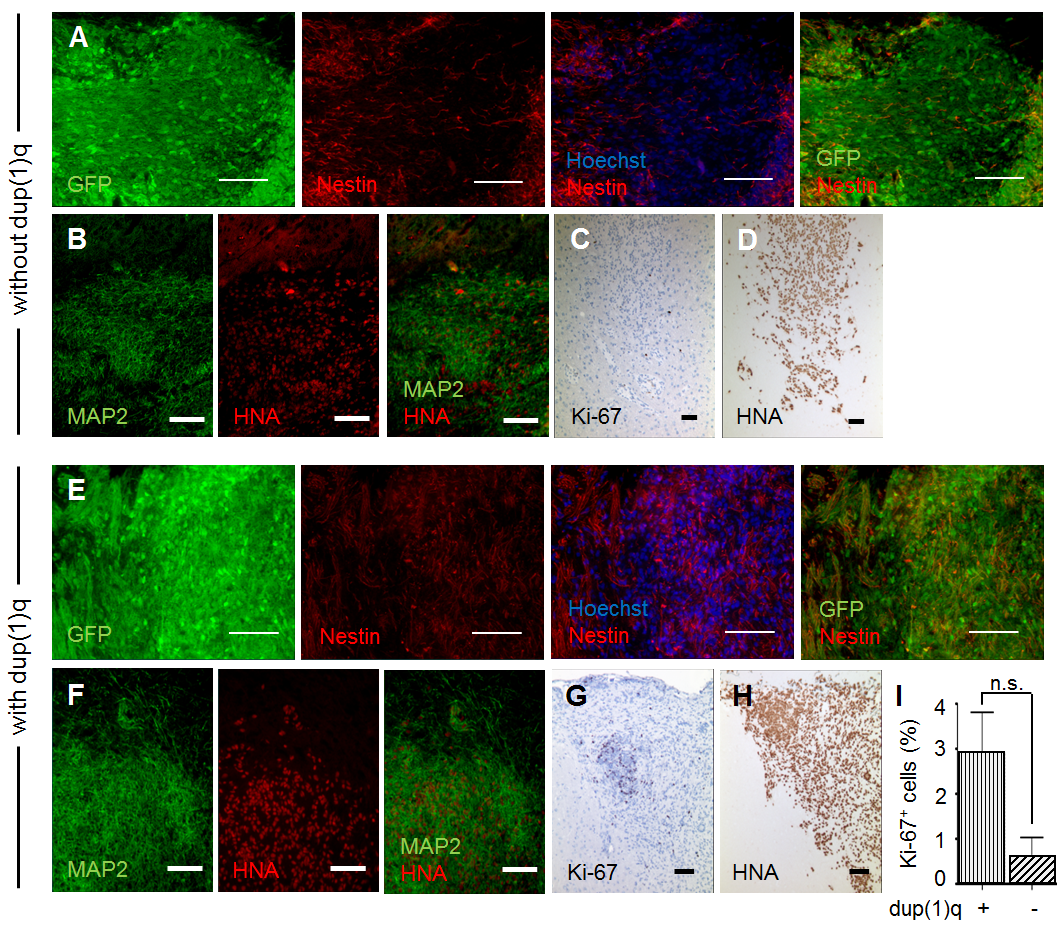
**

**Figure S15. Long-term engraftment of ZFN-NSCs with and without dup(1)q after transplantation into the striatum of immunodeficient rats**. (**A, E**) Immunohistochemical analysis of rat brain sections two months after transplantation with ZFN-NSCs (clone 44) revealed that clearly delineated GFP-expressing cell grafts still persisted at the transplantation site in animals that received NSCs without (**A**) and with dup(1)q (**E**). Some of these cells were also positive for Nestin, which suggested that immature NSCs were still present at that time point in the brain tissue of both groups and had not differentiated into more mature neural lineages. (**B, F**) Staining of engrafted ZFN-NSCs for the neuronal cell marker MAP2 (green) and human nuclear antigen HNA (red) indicates that some transplanted cells differentiated to neurons in both groups. (**C, G**) Assessment of proliferative cell activity in these areas showed that a few Ki-67-positive cells resided in the graft area, which was otherwise rich in HNA-positive human ZFN-NSCs as shown on neighbouring slices (**D, H**). Slices stained with Ki-67 were counterstained with hematoxylin & eosin (**C, G**). (I) The frequency of Ki-67 positive cells was higher in graft areas of animals transplanted with ZFN-NSCs that harboured dup(1)q compared to those that contained NSCs without chromosomal aberrations; however, this difference was not statistically significant (n.s.) because of a very low percentage of dividing cells.

**
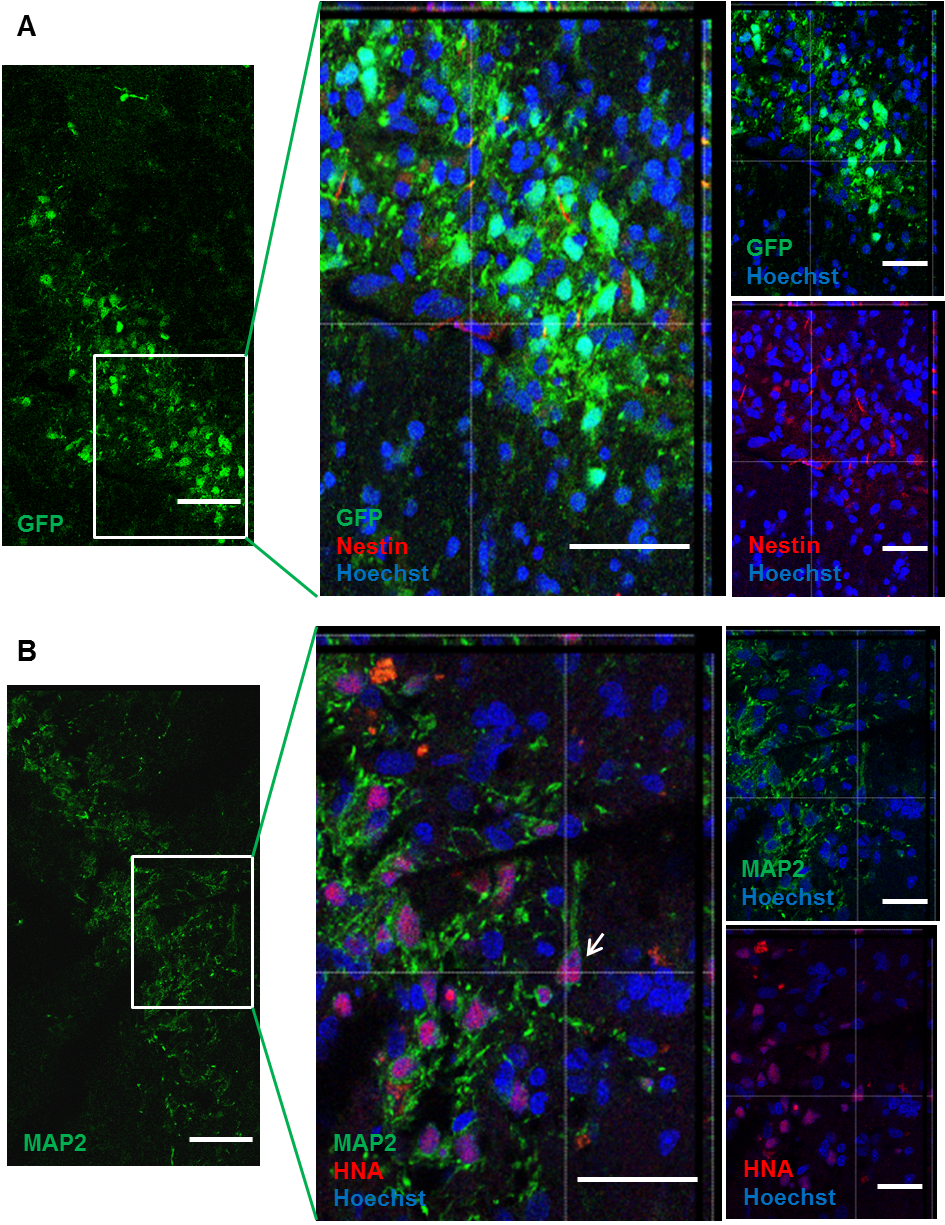
**

**Figure S16. Expression of Nestin and extension of neurites from neurons derived from ZFN-NSCs with dup(1)q in the striatum of the immunodeficient rat brain.** (**A**) Confocal microscopy of tissue slices from rat brain obtained two months after transplantion with ZFN-NSCs (clone 44) carrying dup(1)q stained with antibodies specific for human Nestin (red) and for transgenic GFP (green). (**B**) Immunohistochemical analysis of neighbouring tissue slices with antibodies against neuronal marker MAP2 (green) and human cell marker HNA (red) demonstrated that at two months after transplantation some human ZFN-NSCs differentiated to neurons and integrated into the host tissue (arrows). Scale bars: 100 µm.

**Supplemental Tables**

**Table S1. Primers for cloning and amplification of probes for Southern blotting**

| **Application** | **Sequence, 5’ 🡪 3’** | **Product size (bp)** | **Anneal. temp.**  **(°C)** |
| --- | --- | --- | --- |
| Primers* to prepare In-fusion PCR product for the donor vector | Puro-2A-GFP-infu-F: **GCTTGAATTCTCTAG**AGAAGTTGTGGGGAGGG- GTCG  Puro-2A-GFP-infu-R: **CGAGAATATTTCTAG**AGGCATTAAAGCAGCGT- ATCCA | 3000 | 60 |
| Primers for preparing eGFP probe for Southern blot | Fg: AGCTGACCCTGAAGTTCATC  Rg: TGATATAGACGTTGTGGCTGTTG | 336 | 60 |
| Primers for detection of eGFP expression | P7: CTCGAGCCGGTGAAACAGACTTTGAATTTT  Rg: TGATATAGACGTTGTGGCTGTTG | 550 | 60 |
| Primers for detection of mono- and bi-allelic ZFN-NSC clones | P1: GTGGCTCTGGTTCTGGGTACTTTTATCTG  P2: GGTGTGTCACCAGATAAGGAATCTGCC | 173 bp (intact); 3165 bp inserted clone | 60 |
| PCR to produce a template for sequencing of the 5` side of the AAVS1 integration site | P3: GGCCCTGGCCATTGTCACTT  P4: TCAGATCTAGAGAGCTCATGAC | 2956 | 60 |
| PCR to produce a template for sequencing 3` side of the AAVS1 integration site | P5: TGGTGAGCAGGGCGAGGAG  P6: GGAACGGGGCTCAGTCAGTCTG | 2025 | 60 |

* Nucleotides marked in bold indicate the 15-bp extensions at 5’-end of the primer that are complementary to the ends of the *Xho*I-linearized donor vector. The remaining nucleotides represent gene specific sequences.

### Table S2. Sequencing primers

| **Name** | **Sequence, 5´ 🡪 3´** | **Application** |
| --- | --- | --- |
| Seq5´ F | GGGACCACCTTATATTCCCAGGG | Sequencing of PCR product from P3 and P4 amplification (Forward) |
| Seq5´ R | CCCACCCTCGGGAAAAAGG | Sequencing of PCR product from P3 and P4 amplification (Reverse) |
| Seq3´ F | GTTCAGGGGGAGGTGTGGGAG | Sequencing of PCR product from P5 and P6 amplification (Forward) |
| Seq3´ R | CTCCAGGAAATGGGGGTGTGTC | Sequencing of PCR product from P5 and P6 amplification (Reverse) |

**Table S3.** **PCR primers used for gene expression analyses**

| **Name** | **Ref. seq.** | **Sequence, 5’ 🡪 3’** | **Product size (bp)** | **Anneal. temp. (°C)** |
| --- | --- | --- | --- | --- |
| DNMT3B | NM_  006892.3 | F: GTCGTGCAGGCAGTAGGAAA  R: GCCATTTGTTCTCGGCTCTG | 175 | 60 |
| PIK3C2B | NM_  002646.3 | F: CCACCGCATCCCCATCATCT  R: GAGCTCTTCGGGTCTGCAGGG | 110 | 60 |
| AKT3 | NM_  005465.4 | F: AATGGACAGAAGCTATCCAGGC  R: TGATGGGTTGTAGAGGCATCC | 130 | 60 |
| MDM4 | NM_  001204171.1 | F: GCGACTCATGGAGCTGCCGT  R: CATTTCACCTTGCGCACCTGC | 196 | 60 |
| NOTCH2NLA | NM_  203458.4 | F: TGCAGTGTCGAGATGGCTATG  R: CGGTTCTTCTCACAGGGGTC | 139 | 60 |

**Table S4: Primary antibodies**

| **Antibody** | **Clone** | **Host*** | **Specificity*** | **Supplier** | **Catalogue number** | **Dilution** | **Used in**** |
| --- | --- | --- | --- | --- | --- | --- | --- |
| O4 | O4 | M | H, M, R | Sigma | O7139 | 1/500 | ICC |
| SOX1 | polyclonal | Rb | H, M | Abacam | ab22572 | 1/1000 | ICC |
| PAX6 | polyclonal | Rb | H, M | Abcam | ab5790 | 1/400 | ICC |
| TRA-1-85 | TRA-1-85 | M | H | R&D | MAB3195 | 1/100 | ICC |
| TUJ1 | 2G10 | M | H, M, R | Santa Cruz | sc-80005 | 1/1000 | ICC |
| Nestin | Rat-401 | M | H | Chemicon | MAB353 | 1/200 | ICC, IHC |
| GFAP | G-A-5 | M | H, Pig, R | Sigma | G3893 | 1/500 | ICC, IHC |
| MAP2 | polyclonal | Rb | H, M, R | Santa Cruz | sc 20172 | 1/500 | ICC, IHC |
| HNA | 235-1 | M | H | Abcam | ab191181 | 1/200 | IHC |
| GFP | polyclonal | Rb | GFP | Thermo  Fisher | A11122 | 1/200 | IHC |
| PSA-NCAM | 2-2B | M | H, M, R | Chemicon | MAB5324 | 1/100 | FC |

* H - Human; M - Mouse; R - Rat; Rb - Rabbit.

** ICC - Immunocytochemistry; IHC - Immunohistochemistry; FC - Flow cytometry.

**Table S5: Secondary antibodies**

| **Antibody** | **Host** | **Specificity** | **Supplier** | **Cat. number** | **Dilution** | **Used in*** |
| --- | --- | --- | --- | --- | --- | --- |
| Alexa Fluor 555 | Goat | Rabbit IgG | Thermo  Fisher | A-21428 | 1/1000 | ICC, IHC |
| Alexa Fluor 555 | Goat | Mouse IgG | Thermo  Fisher | A-21422 | 1/1000 | ICC, IHC |
| Normal IgM isotype control | Mouse | - | Santa Cruz | sc-3881 | 1/100 | FC |
| Alexa Fluor 555 | Goat | Mouse IgM | Molecular Probes | A-21426 | 1/1000 | FC |

* ICC - Immunocytochemistry; IHC - Immunohistochemistry; FC - Flow cytometry.

**Supplemental references**

**1.** Prochnow, N., and Schmidt, M. (2004). Spontaneous activity of rat pretectal nuclear complex neurons in vitro. BMC Neurosci *5*, 29.

**2.** Grundken, C., Hanske, J., Wengel, S., Reuter, W., Abdulazim, A., Shestopalov, V.I., Dermietzel, R., Zoidl, G., and Prochnow, N. (2011). Unified patch clamp protocol for the characterization of Pannexin 1 channels in isolated cells and acute brain slices. J Neurosci Methods *199*, 15-25.

**3.** Totonchi, M., Taei, A., Seifinejad, A., Tabebordbar, M., Rassouli, H., Farrokhi, A., Gourabi, H., Aghdami, N., Hosseini-Salekdeh, G., and Baharvand, H. (2010). Feeder- and serum-free establishment and expansion of human induced pluripotent stem cells. Int J Dev Biol *54*, 877–886.

**4.** Koch, P., Opitz, T., Steinbeck, J.A., Ladewig, J., and Brustle, O. (2009). A rosette-type, self-renewing human ES cell-derived neural stem cell with potential for in vitro instruction and synaptic integration. Proc Natl Acad Sci U S A *106*, 3225-3230.
